# Structural Conversion of Aβ_17–42_ Peptides from Disordered Oligomers to U-Shape Protofilaments via Multiple Kinetic Pathways

**DOI:** 10.1371/journal.pcbi.1004258

**Published:** 2015-05-08

**Authors:** Mookyung Cheon, Carol K. Hall, Iksoo Chang

**Affiliations:** 1 Center for Proteome Biophysics, Department of Brain & Cognitive Sciences, Daegu Gyeongbuk Institute of Science and Technology (DGIST), Daegu, Korea; 2 Department of Chemical and Biomolecular Engineering, North Carolina State University, Raleigh, North Carolina, United States of America; University of Virginia, UNITED STATES

## Abstract

Discovering the mechanisms by which proteins aggregate into fibrils is an essential first step in understanding the molecular level processes underlying neurodegenerative diseases such as Alzheimer’s and Parkinson's. The goal of this work is to provide insights into the structural changes that characterize the kinetic pathways by which amyloid-β peptides convert from monomers to oligomers to fibrils. By applying discontinuous molecular dynamics simulations to PRIME20, a force field designed to capture the chemical and physical aspects of protein aggregation, we have been able to trace out the entire aggregation process for a system containing 8 Aβ17–42 peptides. We uncovered two fibrillization mechanisms that govern the structural conversion of Aβ17–42 peptides from disordered oligomers into protofilaments. The first mechanism is monomeric conversion templated by a U-shape oligomeric nucleus into U-shape protofilament. The second mechanism involves a long-lived and on-pathway metastable oligomer with S-shape chains, having a C-terminal turn, en route to the final U-shape protofilament. Oligomers with this C-terminal turn have been regarded in recent experiments as a major contributing element to cell toxicity in Alzheimer’s disease. The internal structures of the U-shape protofilaments from our PRIME20/DMD simulation agree well with those from solid state NMR experiments. The approach presented here offers a simple molecular-level framework to describe protein aggregation in general and to visualize the kinetic evolution of a putative toxic element in Alzheimer’s disease in particular.

## Author Summary

Understanding the mechanisms of protein folding and aggregation is of fundamental importance in elucidating the biological function of proteins and their complex. Many advances have been made in our ability to describe protein folding based both on ideas from biophysics and improvements in supercomputing power, yet realistic simulations of the entire kinetic process of protein aggregation including fibril formation still remain challenging tasks in biophysics and computational biology. This work describes a breakthrough in our ability to simulate the aggregation of proteins on a molecular level and the emergence of the toxic species responsible for the cause of neuro-degenerative diseases such as Alzheimer’s disease. Based on this work, one can now trace the entire aggregation process starting from disordered monomers to meta-stable oligomers to protofilament and then amyloid fibril. This is a significant advance over the current state of the art in both biophysics and computational biology in uncovering the fundamental mechanisms behind the amyloid fibril formation for aggregation-prone proteins.

## Introduction

The aggregation of amyloid β protein (Aβ), the likely cause of Alzheimer's disease, is widely studied via experiment and computational efforts.[1,2] The end product of the Aβ aggregation process is a fibril whose structure depends strongly on the environment and has diverse polymorphic features, although U-shape (β strand—turn- β strand motif) β-sheets’ protofilaments are a consistent theme.[2–7] One of the important goals in the current research is to understand the kinetic mechanism of fibril formation together with the ultimate goal for identifying the toxic species, which are now thought to be early-stage soluble oligomers, and also clarifying their structural and kinetic characters.[8–11] A number of candidates for those toxic oligomers have been suggested, including the paranuclei, pentamers and hexamers of Aβ42 peptides, which are observed *in vitro*, and β-rich structures with exposed hydrophobic residues which are thought to form *in vivo* in the vicinity of bi-lipid membranes.[8,11–16] Several candidate toxic oligomers appear to have a generic structural character such as a bend in the C-terminal near residues G37 and G38.[8,14,17] In fact Pande and coworkers have shown that designing a turn into the C-terminal by mutation enhances the stability of the oligomers which, in their experiment, appear to be off-pathway.[17] In addition Smith and coworkers observed S-shape monomers (from K16 to A42) containing a C-terminal turn within disc-shape pentamers and found these oligomers to be toxic.[8] Teplow and coworkers also detected toxicity in Aβ oligomers containing a C-terminal turn designed by mutation.[14] Despite some advances recently in our knowledge on the fibrillization process and the identity of toxic species, detailed molecular-level descriptions of the structural conversion of Aβ monomers to early stage oligomers to potentially toxic oligomers to protofilaments are not yet available. Knowledge of the oligomerization and structural conversion of Aβ peptides to proto-fibrils at the atomic scale would allow us to ascertain how the toxic species emerge and how they achieve meta-stability.

The focus of this paper is Aβ17–42, a 26-residue C-terminal fragment of Aβ42, the peptide whose aggregation is most strongly linked to Alzheimer’s disease. Aβ17–42 is produced from the cleavage of amyloid precursor protein by α- and γ-secretases and is observed in amyloid plaques which are composed of amyloid fibrils.[18] It has been suggested that the Aβ17–42 structures form U-shape protofilaments similar those for Aβ40 or Aβ42, which is supported by computational stability study.[19] Since Aβ17–42 is a key fragment of Aβ42, the formation of its U-shape protofilament is likely to be very similar to that of its longer parent Aβ42. Justification for this idea is that Aβ17–42 contains the two hydrophobic stretches that dominate the aggregation and fibrillization of Aβ42 as well as the turn region. In addition, it appears that the N-terminal Aβ residues 1–10 or 1–16 do not participate in the rigid portions of the U-shape protofilament in synthetic fibrils observed by the groups of Tycko, Riek and Bertini,[3,4,20,21] although they do participate in the tubular shape protofilaments observed by Zhang et al. and Miller et al.[5,22,23] serving as arms that form a sheath surrounding the hollow core structures. They also participate in the ordered fibril structures derived from Alzheimer’s brain tissue.[24] Aβ42 is a more toxic peptide than Aβ40 or Aβ17–42, which means that the two C-terminal residues and the rather flexible N-terminal residues likely play an important role in amyloidogenesis and toxicity, as is supported by mutagenesis and bioinformatics studies.[25–27] Since it would be extremely difficult to simulate spontaneous fibril formation of full length Aβ42 given current computational constraints, we focus here on the role of the C-terminal residues and their turns. Restricting our attention to Aβ17–42 also makes it easier to watch the spontaneous U-shape conformation form without the encumbrances that would occur in the presence of the highly flexible N-terminal residues.

Molecular *in-silico* description of spontaneous fibril formation by Aβ40, Aβ42 and even Aβ17–42 based on all-atom or coarse-grained models is still extremely challenging due to our inability to capture the multi-scale nature of the force-field, the very long time scales for fibrillation (much longer than that for protein folding) and the variety of polymorphic structures observed with different backbone orientations, protofilament conformations, and protofilament stacking arrangements.[6] All atom simulations examining the kinetic stability of Aβ17–42 peptides in preformed stacked fibrillar structures and annular oligomeric structures characteristic of ion-channels have been conducted.[19,28–30] Computational studies of the spontaneous oligomerization of Aβ40 and Aβ42 using coarse-grained models have been performed.[31,32] Hybrid combinations of all-atom and coarse-grained simulations of Aβ40, Aβ42 and Aβ17–42 have been performed on 2 and 3-peptide systems.[33,34] Fibril elongation by monomer addition to a preformed Aβ17–42 fibrillar structure has been simulated for very long times (~1.3ms) by a hybrid resolution molecular dynamics.[35] The extensive model and kinetic network analysis reveals atomistic details of a monomer participating in the dock-and-lock mechanism[36], thereby explaining unidirectional fibril growth. This work shows that addition and reorganization near a preformed structure containing as little as one monomer needs extensive simulation time. All-atom simulations with Aβ42 dimer and inhibitors have been performed to understand the inhibitory mechanism for oligomerization.[37] However, the entire fibrillation pathways starting from randomly denatured structures progressing through the formation of oligomeric intermediates and leading to formation of the U-shape fibril structures, to be consistent with those from experiments, are not accessible yet.

In this study, we apply discontinuous molecular dynamics (DMD) simulations in conjunction with the PRIME20 force field[38–40] to simulate fibrillation of systems containing 8 Aβ17–42 peptides initially starting from their randomly disordered conformations. Fibril structures with U-shape β-sheets are constructed successfully in our DMD simulations, which then provide structural information during the entire kinetic process of aggregation. Among the many simulations performed at various temperatures, we focus on on-pathway trajectories which provide excellent fibril structures consistent with those suggested in the experiments.[8,20] Two different mechanisms for structural conversion from randomly disordered conformations to protofilaments emerge from different pathways: (1) one-by-one monomeric conversion to a fibrillar structure, and (2) slow conversion through a meta-stable oligomer with “S”-shape conformations containing a C-terminal outward turn to a final fibrillar structure. The “S”-shape conformations are postulated to be a key component of toxic oligomers, based on results of our simulations and others’ experiments.[8,14,17]

## Results

### Dependence on system size

We performed preliminary simulations with different numbers of Aβ17–42 peptide chains (NC) i.e. NC = 1, 2, 4, 5, 6, 8, 10, 12. Representative structures for NC = 1, 2, 4, 5, 6 are shown in S1 Fig of Supporting Information (SI). For NC = 1, a disordered monomer is observed (S1A Fig). For NC = 2, two separated disordered monomers are seen in most simulations and partially ordered dimers are rarely observed (S1B Fig). For NC = 4, a tetramer is easily formed but β-helix structures are observed in most simulations (S1C Fig), which means oligomerization occurs but structural conversion toward U-shape conformation is not accessible yet. For NC = 5, we observe both β-helix and U-shape conformations (S1D Fig). For NC = 6, we observe U-shape conformations more frequently (S1E Fig). Hence NC = 5 or NC = 6 can be considered to be the critical nucleus size for conformational conversion for Aβ17–42 peptide under present simulation conditions. For NC = 8, we also observe U-shape conformations, but for NC = 10 and 12, we observe partial U-shape conformations which means a longer simulation time or more thermal fluctuations are needed to convert toward highly ordered protofilaments. Hence NC = 8 is the best system size for studying both structural conversion from disordered structures and growth mechanisms toward protofilaments within present accessible simulations. The detailed results on the various structures and analysis of the system size dependence of Aβ17–42 peptides will be presented in a future paper.

### Fibril structure of Aβ17–42 with 8 chains

DMD/ PRIME20 simulations were performed on an 8-chain system of Aβ17–42 peptides starting from a random configuration. The original PRIME20 force field[38,40] was augmented to include the parallel preference constraints for hydrogen bond angles, an enhanced salt-bridge interaction (ε_KE_ = 0.4ε_HB_) between K28 and D23 residues where ε_HB_ is the hydrogen bonding energy between NH and C = O, and double well potentials for all of the side-chain pair interactions. These modifications to PRIME20 significantly reduce the complexity associated with sampling the energy landscape, which contains a variety of polymorphic conformations as has been shown in computational studies and in experiments.[4–6,19,41–43] We simulated at reduced temperatures, T* = (k_B_T/ε_HB_) in the range from 0.19 to 0.205, which is near the “fibrillization temperature”, the temperature above which fibrils cease to form spontaneously; above this temperature the peptides equilibrate as random monomers and small disordered oligomers without β-sheet content. Each simulation is started at high temperature T* = 0.5 with different random seeds from eight separated denatured monomers without any inter-peptide contacts in a periodic box (L^3^ = (160Å)^3^ corresponding to 1.7mM) to ensure a randomly disordered initial configuration. The system is slowly cooled from T* = 0.50 to final temperature over the first 8 billion collisions (t* = 788) and thereafter remains at constant reduced temperature.

**Fig 1:**
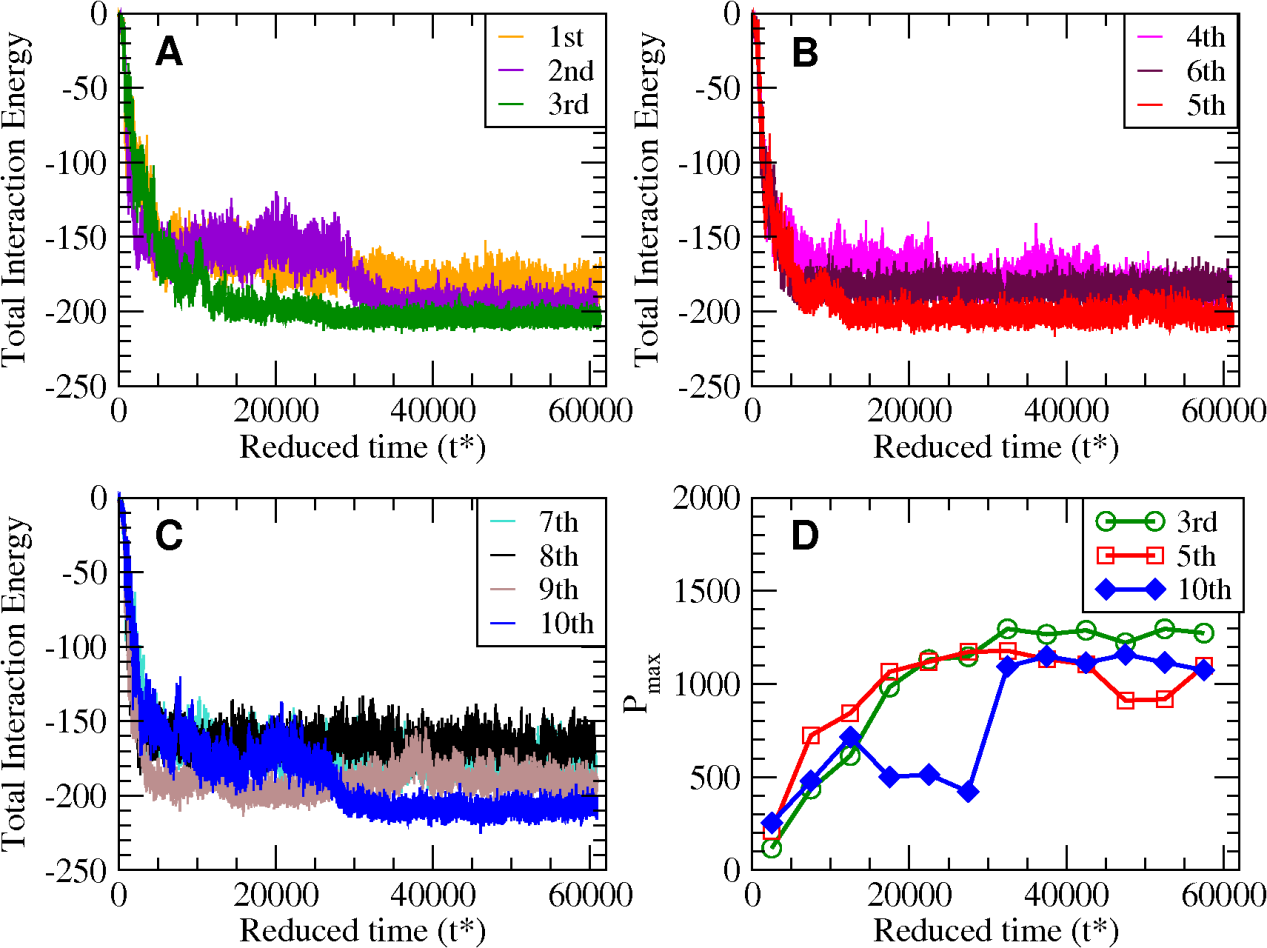
Time evolution of the interaction energy. The total interaction energy in units of ɛ_HB_ for (A) 1st, 2nd, 3rd, (B) 4th, 5th, 6th, (C) 7th, 8th, 9th, 10th trajectories. The 3rd (green), 5th (red), 10th (blue) trajectories show lower energies than the others. (D) P_max_ (max population) within each Δt* = 5000 interval which is defined in text.

**Fig 2:**
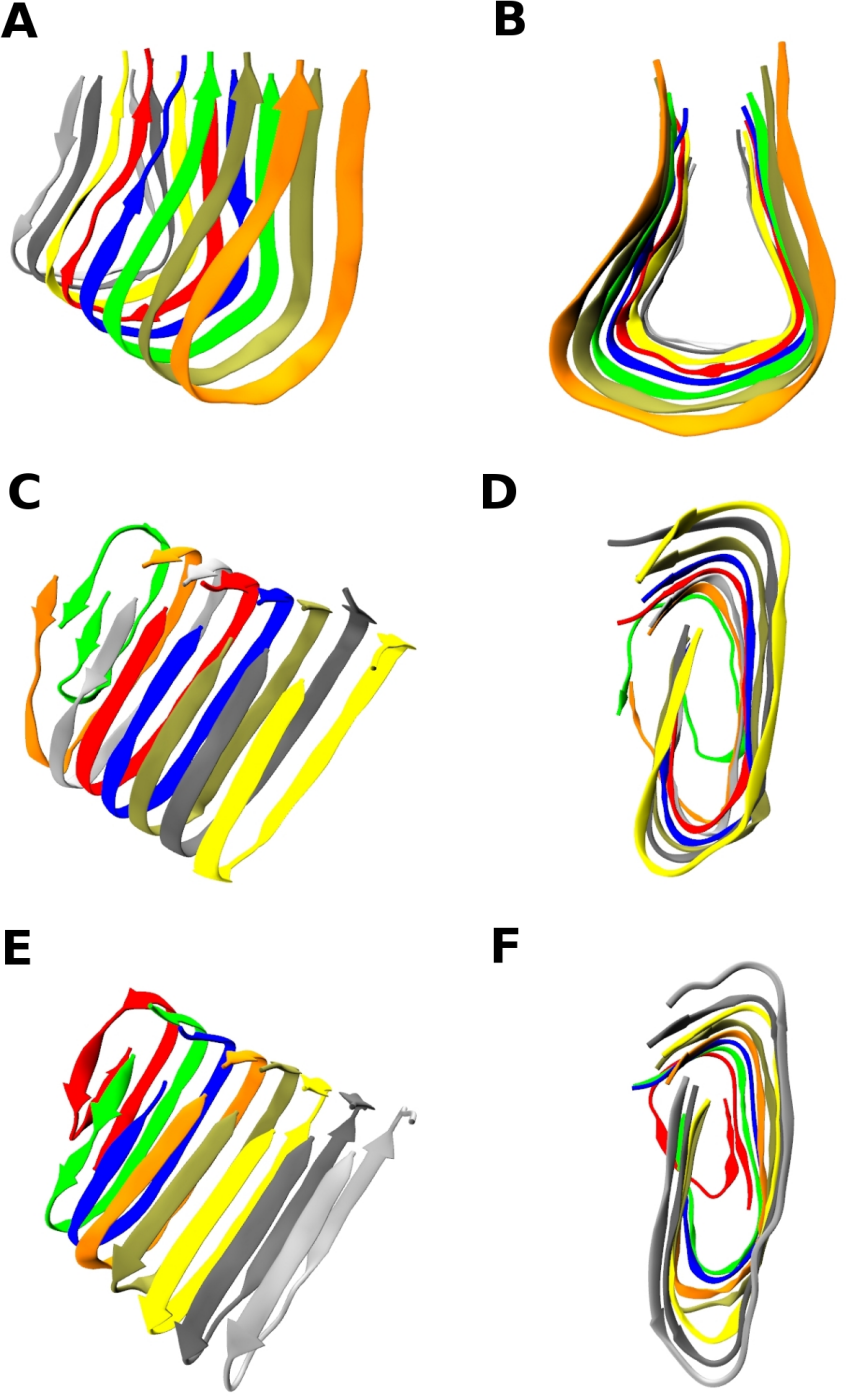
Three structures with the lowest energy. Three selected final structures of protofilaments for 8 Aβ17–42 peptides among 10 independent runs at T* = 0.20. Structures for (A) (B) the 3rd run, (C) (D) the 5th run, (E) (F) the 10th run after 668 billion collisions (reduced time t* = t/σ(k_B_T/m)^1/2^ ≈ 61,000). Figs (B) (D) (F) are the front views along the fibril axis and (A) (C) (E) are the side views, respectively.

Fig 1A–1C shows the total interaction energy versus time for ten long (668 billion collisions or t* = t/σ(k_B_T/m)^1/2^ ≈ 61,000) independent simulations at T* = 0.20, where σ and m are the united N-H sphere diameter and mass respectively. The 3^rd^(green), 5^th^(red) and 10^th^(blue) trajectories have the lowest total interaction energy and hence are most thermodynamically stable according to the PRIME20 force field. Fig 2 shows these three configurations that are nicely-formed 8-chain fibrillar (protofilament) structures. The other seven structures are shown in S2 Fig; they are all partially ordered with high β-sheet content. Interestingly the structures are quite varied even though the same simulation conditions were applied in each run. The structure in Fig 2A and 2B (from 3rd run) shows all eight peptides adopting a bent shape with in-register β-sheets but the loop has a triangular shape rather than a U-shape. Two of the chains (silver and gray) in Fig 2A are antiparallel to the other six chains, which means that the turning points between the two β-sheets are mismatched. S3A and S3B Fig shows the location of the glycines on these structures. While the only glycine residue participating in the U-turn in S3C–S3F Fig is G25(blue), both G25(blue) and G33(red) participate in the U-turn in S3A and S3B Fig; they facilitate the formation of the two turns that appear on the anti-parallel β-sheets (cyan and gray in S3A and S3B Fig), encouraging the protofilament to form a triangular shape. This structure is very similar to a structure suggested by the Wetzel group in their early studies of Aβ with the slight differences in the location of the turns.[44,45] The structures resulting from the fifth and the tenth runs shown in Fig 2C–2F are highly organized U-shape β-sheet structures that have hydrogen bonds between neighboring chains in adjacent strands within the sheet. This structure has the U-shape characteristic of those found via experiment by Lührs et al and Petkova et al.[20,21]

**Fig 3:**
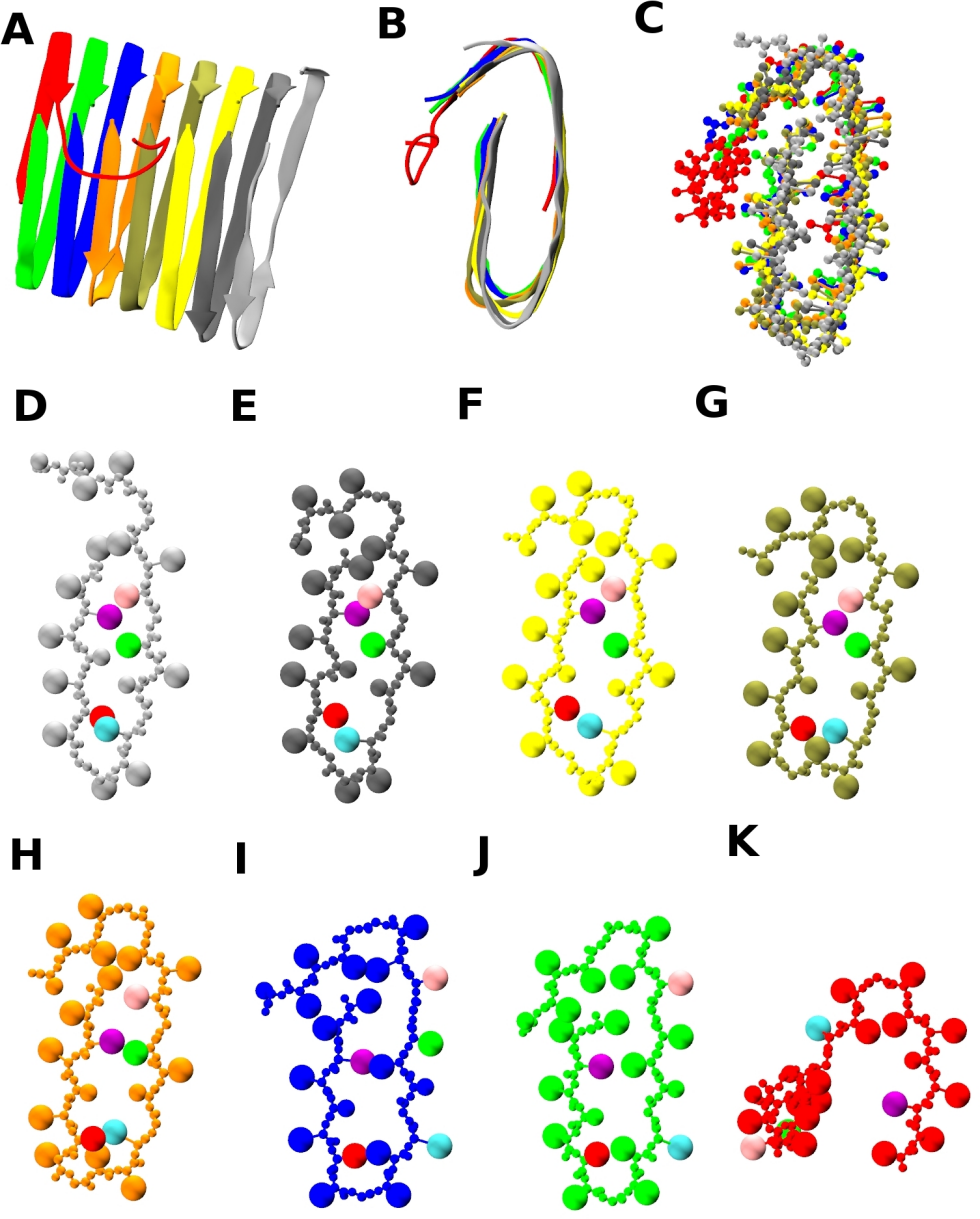
Salt-bridge and hydrophobic interactions for the 10^th^ run. (A) Structure at 568 billion collision (t*≈52,000) for the 10^th^ run. (B)(C) Fibril axis view with ribbon diagram or with side-chain spheres. (D)~(K) Fibril axis views for each chain showing side-chain spheres; F19(purple), D23(red), K28(cyan), I32(green) and L34(pink sphere). Figs (D)~(H) have salt-bridge pairs (D23-K28) and hydrophobic interactions between I32, L34 and F19; the rest do not.

Fig 3A–3C shows ribbon and ball-and-stick snapshots of the structures (including the positions of the side chains for each of the eight chains in the structure) for the 10th run, enabling the visualization of the turn region, the salt-bridge interaction, and the residues which interact hydrophobically inside the U-shape β-sheet. Fig 3D–3K show the positions and identities of the side chains in each of the eight chains from Fig 3A–3C. Although the D23(red) and K28(cyan) spheres in Fig 3D–3H are inside the U-shape β-sheet, the K28(cyan) spheres in Fig 3I and 3J are outside, indicating that salt-bridges are formed for the first five chains but absent for the last three chains. The structure for the 5th run in Fig 2C and 2D has fewer salt-bridges; there are only two D23 and K28 pairs (silver and orange chains) inside the U-shape β-sheet as shown in S4 Fig. Hence we see the D23-K28 salt bridge is not easily found kinetically in our simulations even though the D23-K28 pair interaction is enhanced. This is not surprising in the light of the observation of weaker D23-K28 coupling in quiescent conditioned fibrils than in agitated conditioned fibrils (presuming here that agitation enhances the approach to fibrillization).[43]

For the 10th run, five chains in Fig 3D–3H show hydrophobic interaction between F19(purple), I32(green), L34(pink) residues inside the U which is consistent with the Tycko group experimental model[20] and other recent experiments by Smith and coworkers[8]. The structure of the turn region from V24 to N27 for the highly fibrillized chains in Fig 3D–3H and the pattern of the hydrophobic side chains on the inside of the U-shape β-sheet are consistent with the Tycko model[20] and the Ma-Nussinov model[46], but is slightly different from the Lührs model[21], which has the turn running from S26 to A30. An unexpected feature in the Fig 2C–2F structure is the existence of a second turn near the C-terminal; this has been suggested in some experiments as being characteristic of toxic oligomers and has been observed in simulations.[8,14,17,31] This tendency to turn is likely enhanced in our simulations due to our omission of the N-terminal, Aβ1–16, which frees up the hydrophobic L17 residue to interact with C-terminal I41 and A42 residues.

### Different structural conversion pathways

**Fig 4:**
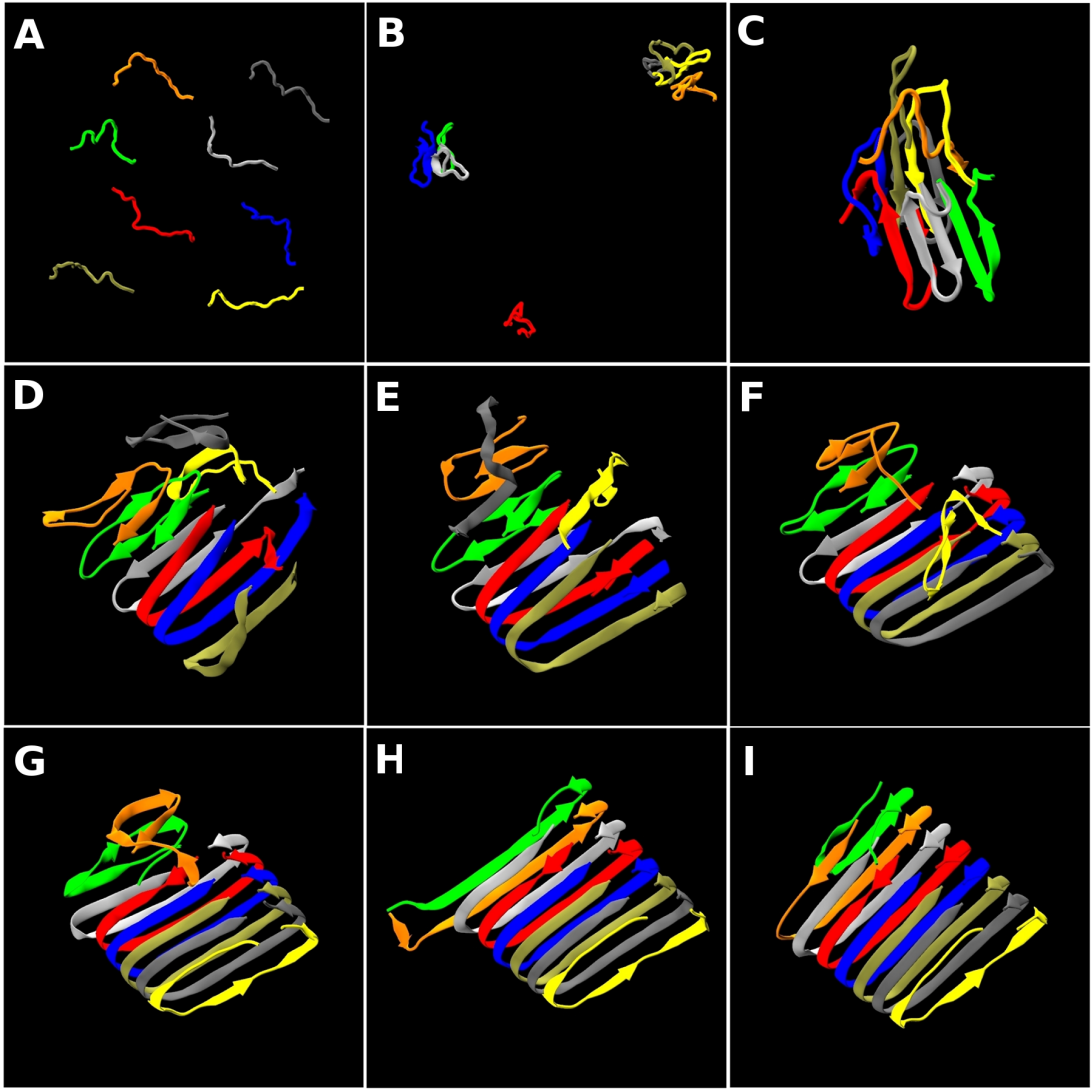
Snapshots for the 5^th^ run. The time evolution of the structure for the 5^th^ run at T* = 0.20 in Fig 1C and 1D. Snapshots are taken at (A) t* = 5, (B) 1244, (C) 2608, (D) 3656, (E) 4233, (F) 5442, (G) 6086, (H) 10454, (I) 11063. See S1 Video. The β-strand contents measured by the STRIDE program are (A) 0%, (B) 12%, (C) 26%, (D) 50%, (E) 48%, (F) 66%, (G) 64%, (H) 74%, (I) 75%. The α-helix content is insignificant in these structures and the remaining portions are coil and turns.

We traced out the time evolution of the structure for the best organized fibrils—the 5th run and the 10th run structures in Fig 2C–2F. The trajectories are presented as movie files in S1 and S2 Videos. Fig 4 shows nine snapshots from the 5th run whose final structure is shown in Fig 2C–2D taken at t* = (A) 5, (B) 1244, (C) 2608, (D) 3656, (E) 4233, (F) 5442, (G) 6086, (H) 10454, (I) 11063 after slowly cooling a configuration of random coils from T* = 0.50 to T* = 0.20 over the course of the first 8 billion collisions (t* = 788). At first we just observe random coil structures (Fig 4A). Early snapshots including those in the slow cooling stage are shown in S5A–S5B Fig. Small disordered oligomers then start to form (Fig 4B) and these merge with monomers into one large oligomer by t* = 2608 (Fig 4C) which has some β-sheet character but no U-shape β-sheets. By t* = 3656, two peptides (red & blue chains) have re-arranged to form U-shape β-sheets (Fig 4D) and by t* = 4233 the tan chain joins the U-shape and the C-terminal residues curve inward (Fig 4E). The gray, yellow and silver chains sequentially join the U-shape at t* = 4233, 5442 and 6086 (Fig 4F and 4G and 4H). Finally the orange chain joins the U-shape and we observe seven peptides forming fibril-like structure by t* = 11063 (Fig 4I). The green chain remains flexible till the end of our simulations. Thus it is apparent that the small U-shape β-sheets in Fig 4D serve as a nucleus driving further fibril growth by conformationally changing the attached monomers on a pre-existing proto-filament. This one-by-one structural conversion process is relatively fast once the U-shape nucleus forms.

**Fig 5:**
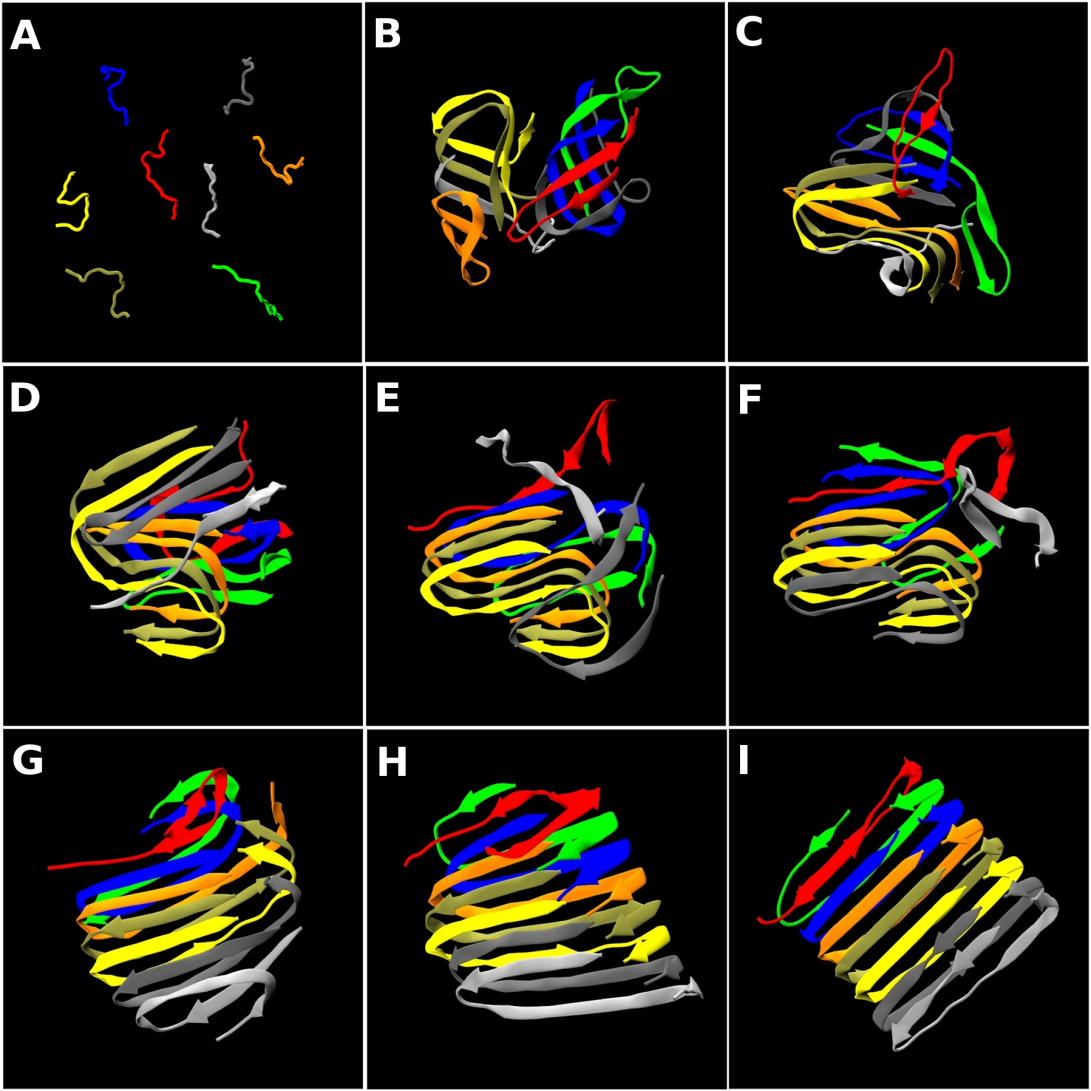
Snapshots for the 10^th^ run. The time evolution of the structure for the 10^th^ run at T* = 0.20 in Fig 1E and 1F Snapshots are taken at (A) t* = 5, (B) 2772, (C) 6623, (D) 19100, (E) 20142, (F) 23060, (G) 26371, (H) 28120, (I) 34039. See S2 Video. The β-strand contents measured by the STRIDE program are (A) 0%, (B) 55%, (C) 58%, (D) 59%, (E) 44%, (F) 50%, (G) 66%, (H) 76%, (I) 74%. The α-helix content is insignificant and the remaining portions are coil and turns.

Fig 5 shows nine snapshots for the 10th run whose final structure is shown in Fig 2E and 2F taken at t* = (A) 5, (B) 2772, (C) 6623, (D) 19100, (E) 20142, (F) 23060, (G) 26371, (H) 28120, (I) 34039. The starting configuration (Fig 5A) is a random distribution of random coils. Early snapshots are shown in S5C–S5E Fig. Small disordered oligomers are observed (S5E Fig). By t* = 2772 one large disordered oligomer has formed (Fig 5B). By t* = 6623, two peptides (yellow & tan chains) form in-register β-sheets with a partially-attached orange chain (Fig 5C). Interestingly, the C-terminal residues curve outward, forming “S-shape” β-sheets. This oligomer with its partial S-shape is meta-stable and remains for a very long time till t* = 19100 (Fig 5D). The orange and gray chains join the S-shape sequentially at t* = 20142 (Fig 5E) and t* = 23060 (Fig 5F), respectively. Thereafter the C-terminal residues begin to change and curve inward forming a U-shape at t* = 26371 (Fig 5G). The silver and blue chains join the U-shape by t* = 28120 (Fig 5H). Finally we observe a nice protofilament-like fibril at t* = 34039 (Fig 5I). The evolution of this fibrillar structure from the disordered oligomer that precedes it is especially interesting. Fig 1C (blue line) confirms that this meta-stable oligomer undergoes structural conversion between t* = 26,000 and 34,000 where the potential energy undergoes a relatively rapid decrease after having been constant for a long time (t* = 10,000 and 24,000). The rapid change in total interaction energy in Fig 1C corresponds to the structural conversion of C-terminal residues from S-shape to U-shape and consecutive monomeric conversion upon joining the U-shape.

Having identified the equilibrated U-shape protofilament together with the long-lived meta-stable oligomer with S-shape conformations, we estimate the relative stability of the various structures that occur in the simulations. We introduce P_max_ (max population) which is defined as the population of configurations, within each Δt* = 5,000 interval along the trajectory, whose total interaction energy is between E_max_—4ε_HB_ and E_max_ + 4ε_HB_, where E_max_ is the total interaction energy of a configuration with the maximum population. Hence P_max_ tells us how often the most populated structures emerged in a given time interval Δt*. It becomes a free-energy-like quantity if we transform it by—*k*_*B*_ *T* log(P_max_). Plots of P_max_ versus reduced time are shown in Fig 1D. For the 3rd (green circle) and the 5th (red square) trajectories, we observe the monotonic increase and saturation in P_max_ as the nice fibril structures are reached. However the 10th trajectory (blue diamond) shows a sub-maximal peak in the population between t* = 7,500 and 17,500 which indicates the emergence of meta-stable structures with S-shape conformations in Fig 5C (t* = 6623) and Fig 5D (t* = 19100). Rapid change in P_max_ is also observed between t* = 27,500 and t* = 32,500. The changes in this free-energy-like quantity are one of the indications for the meta-stability of the oligomers with S-shape conformations. The fact that the oligomer with S-shape chains has such a long lifetime is consistent with the experimental observation that designing in such a conformation via mutation stabilizes Aβ oligomers.[17] The outward turn of the C-terminal residues was also suggested by Smith and coworkers as being characteristic of the toxic Aβ42 oligomers that they observe at low temperature, which converts to U-shape fibrils by increasing temperature.[8] Here we demonstrate at the molecular level that the S shape undergoes a structural conversion to a U shape and hence a nice fibril structure. One difference between our results and the experimental findings of Smith and coworkers is that their S-shape monomer exists within a disc-shape pentamer, not a partial β-sheet structure as we observe here. One possibility is that the fibrillation pathway that Smith and coworkers observed might have included formation of the S-shape β-sheet as an intermediate step between the disc-shape pentamer and the protofilament. This seems to be plausible to us given that they had to raise the temperature to get to the protofilament state and this could have imparted sufficient kinetic energy to transform the disc-shape pentamer to the lower energy S-shape β-sheet.

We analyzed our data to further explore the possible meta-stability of intermediate oligomers with S-shape conformations by performing all-atom simulations for these configurations and final fibrillar structures. All-atom PDBs were generated based on snapshots of Fig 4C–4I and Fig 5C–5I and all-atom molecular dynamics with AMBER/ff99SB force field and explicit solvent TIP3P water were performed for 10ns at 298K. Twelve independent simulations for each initial structure among the fourteen different snapshots were run with different initial random seeds to measure observables related to stability such as system energy, binding energy (intermolecular energy), and RMSF (backbone atomic positional fluctuations), which are shown in S6 Fig. While the highly ordered structures (G, H, I) for the 5th run have lower energy (S6A Fig), the meta-stable intermediates (C, D) and final fibrillar structure (I) for the 10th run show similar energy levels (S6B Fig). However the highly ordered structures (I) have strong binding energy (S6C–S6D Fig) for both runs. Interestingly snapshot E (t* = 20142) from the 10th run, which is just before rapid structural conversion and is corresponding to high energy in Fig 1C and less β-strand content (Fig 5 caption), also has high system energy (S6B Fig), high binding energy (S6D Fig) and high RMSF (S6F Fig). This observation indicates that the intermediates (C and D) with S-chain conformations are meta-stable, consistent with our explanations for the results Figs 1D and 5. We used RMSF instead of RMSD since the latter shows too much fluctuation in our atomistic simulations, losing discrimination power. The RMSF sheds light on the meta-stability of snapshots C, D and the low stability of E for the 10th run. Hence we reconfirmed our observation of the meta-stability of intermediate oligomers with S-shape conformations by all-atom molecular dynamics.

**Fig 6:**
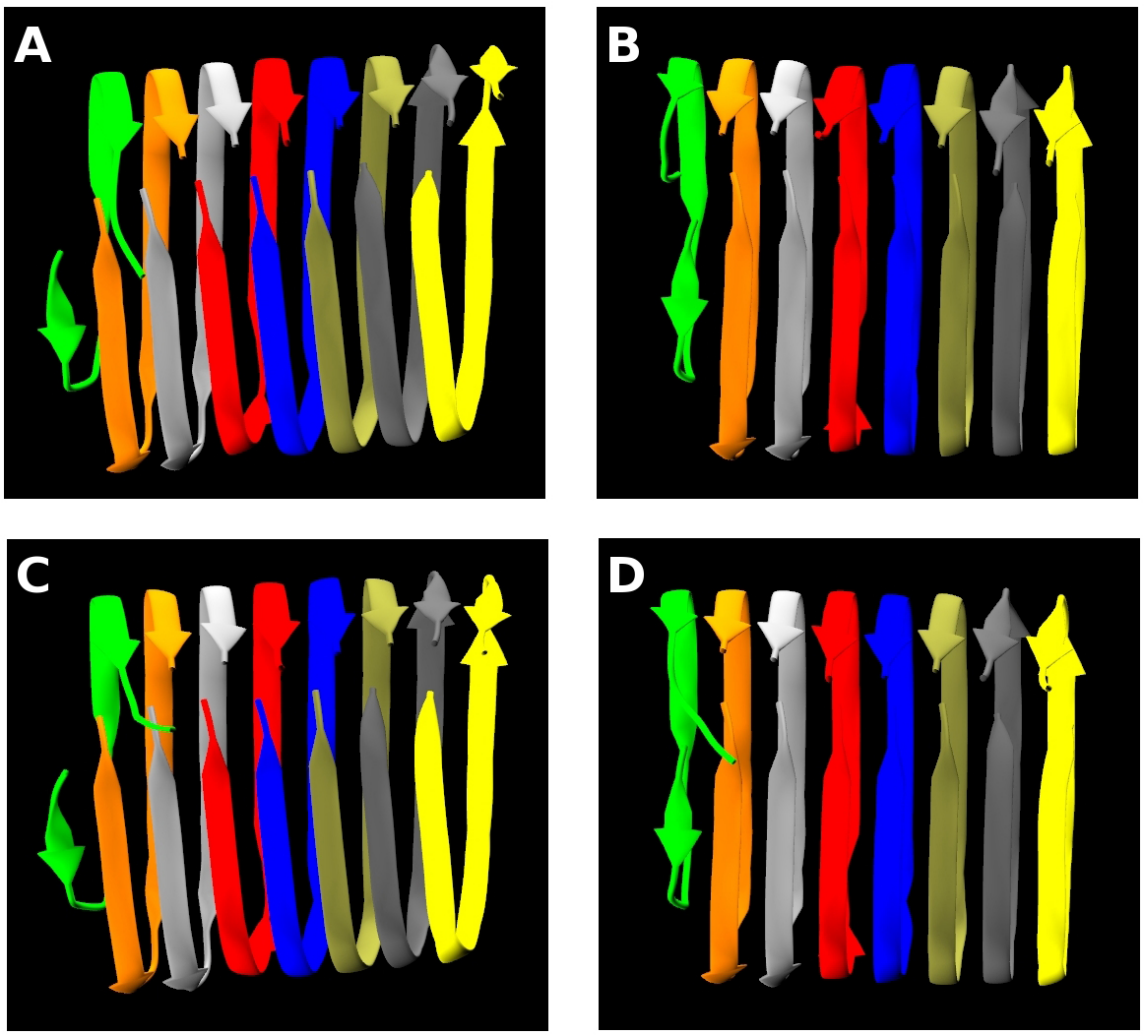
Constant structural switching between intra and inter-chain interactions. Snapshots showing the structural change from inter- to intra-chain association for the 5th run at reduced times (A) t* = 34032 (B) 34059 (C) 34096 (D) 34310 as shown in S3 Video.

Another interesting observation in our PRIME20/DMD simulations has to do with the pattern of side-chain interactions between one side of the U and the other. Fig 6 shows four snapshots for the 5th run at four different times (t* = 34032, 34059, 34096, 34310) selected from the time period t* = 33500 to 42736 shown in S3 Video. In Fig 6A and 6C, the N-terminal β-sheet and the C-terminal β-sheet are slightly tilted with respect to each other so as to form inter-molecular side-chain interactions, similar to those observed in experiments.[20,21] However in Fig 6B and 6D, the N-terminal β-sheet and the C-terminal β-sheet are not tilted, and form intra-molecular side-chain interactions. The surprising result here is that inter-chain interactions which stabilize the U-shape β-sheet structure constantly change to intra-chain interactions and vice versa over the course of our simulations as is clearly shown in S3 Video. It is interesting that we see two polymorphic conformations simultaneously in fibril formation. This observation may be due to finite size effects associated with having only 8 chains; in a larger system one fixed conformation may be stabilized as the structure grows.

Although we focused our discussion of structural conversion on two trajectories at a specific temperature T* = 0.20 among many independent runs, we actually performed DMD simulations at six other temperatures T* = 0.19, 0.195, 0.198, 0.20, 0.202 and 0.205. S7 and S8 Figs show final structures at T* = 0.198 and 0.202 respectively. We observed a triangular shape in S7G Fig, U-shape in S8H Fig, S-shape in S8F Fig, and many partial U-shape or β–helix structures. However we observe only random monomers and disordered small oligomers at T* = 0.205, which means that it is above the fibrillation temperature, and only partially ordered structures at T* = 0.19 and 0.195.

### Structural analysis for each residue

**Fig 7:**
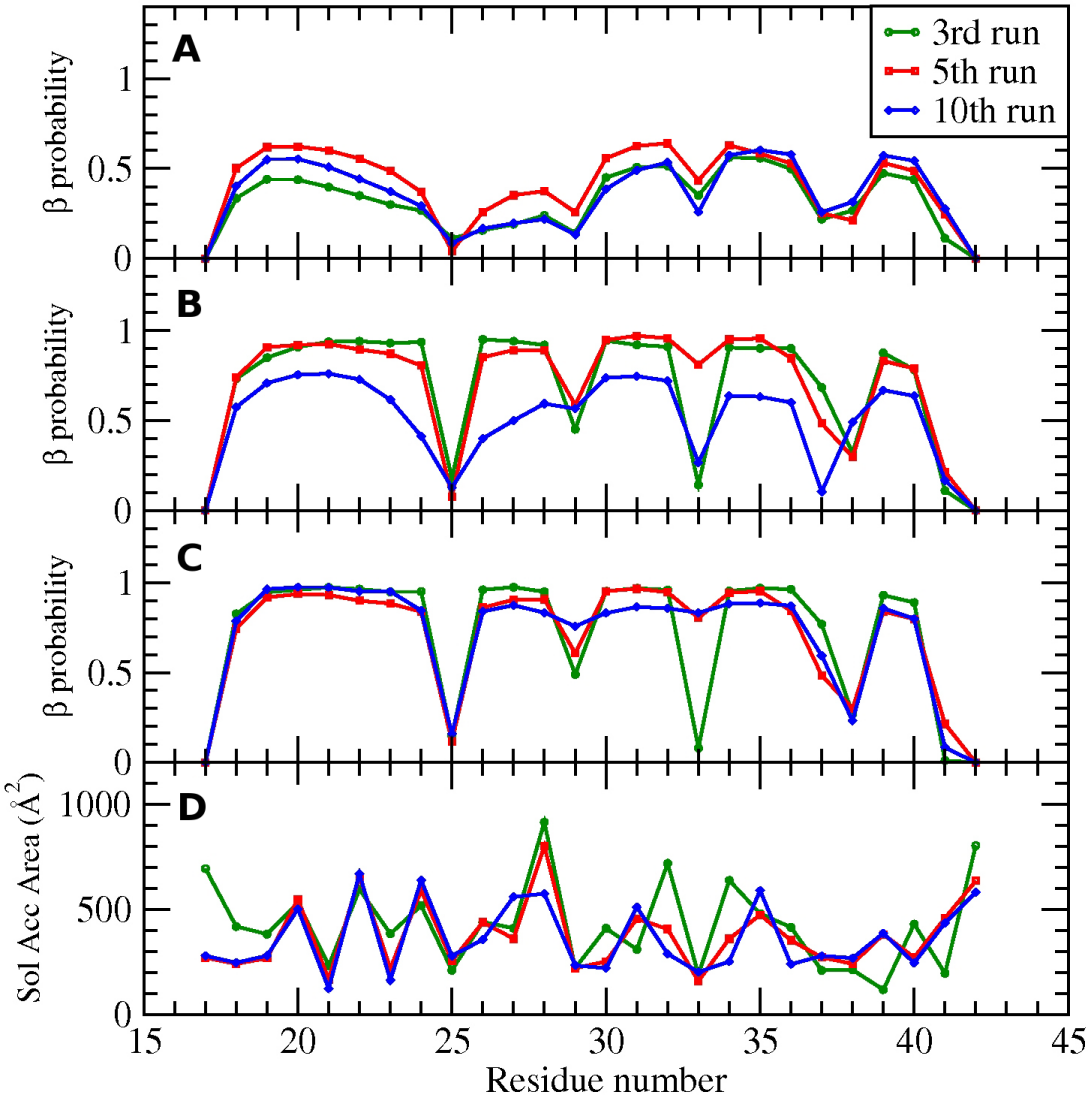
β–strand content and solvent accessible area. Probability that each residue is in β-strand conformation. Data are averaged over three time windows (A) t* = 2153~6727, (B) 19648~24266 and (C) 38120~42736. (D) Solvent accessible area for each residue. Data are averaged over time window (t* = 38120~42736).

The structural and temporal features of our simulations were analyzed using two different methods. In the first method, the β-strand content for each residue was calculated during three time windows in the simulation. The analysis was applied to the 3rd, 5th and 10th runs, all of which led to fibril-like structures. To identify the β-strand content for each residue, we calculated the dihedral angles (φ and ψ) and used the STRIDE program[47] which identifies the secondary structure of each residue over the simulation trajectory. Trajectories were collected and averaged over three time windows: very early stage (t* = 2153~6727) in Fig 7A, middle stage (19648~24266) in Fig 7B and late stage (38120~42736) stages in Fig 7C, to gauge how the β-strands develop over time. The two hydrophobic regions (V18-V24 and A30-V36) develop β-strands starting from the earliest stage and continue to have high β-strand content through the late stage. During the middle stage where the 10th run is still a meta-stable oligomer with S-shape chains, the β–strand content for the 10th run (blue line) is not fully developed yet. At late stage, the 5th and 10th runs clearly show turn regions (V24-S26 and G37-G38). The 3rd run shows another turn region near G33 which is what makes for the triangular shape as shown in Fig 2A and 2B. Those portions of the chain that easily transform to β-strands play an important role in fibril formation because they readily form β-sheets whose protruding side-chains provide opportunities for stacking interactions leading to a cross-β spine.

In the second analysis method, the solvent accessible surface area (SASA) of each residue is measured during the late time window (t* = 38120~42736) shown in Fig 7D. These values are estimated using the STRIDE program which we have further tailored to accommodate the four-sphere PRIME geometry. The zigzag pattern that is observed along the sequence is an expected consequence of the alternating side-chain patterns on the β-strands. Roughly speaking, the N-termini in the 5th and 10th runs are more exposed than the C-terminal, which is consistent with other coarse-grained simulations.[34] This indicates that the hydrophobic residues in C-terminal drive themselves to be buried as is known to occur in oligomers or fibril structures.[4,8] As discussed in the previous section, the 10th run (blue line) shows results that are the most consistent with experiments, having small SASA values at D23, K28, F19, I32 and L34, which are characterized by an internal salt-bridge and hydrophobic interactions.[8,20] To check if our SASA results were an artifact of using the PRIME20 reduced 4-sphere protein representation, we generated all-atom based PDB files by applying the MODELLER program to our PRIME model trajectories and ran the STRIDE program again. The SASA pattern for the all atom structures was similar to the 4-sphere result.

Analysis of the data presented in Fig 7 and that of the snapshots presented in Figs 4 and 5 gives us a molecular-level picture of how disordered oligomers undergo structural conversion towards fibril structure. At an early stage, disordered oligomers are formed with exposed N-terminal and buried C-terminal sites, probably due to having more hydrophobic residues at the C-terminal. The two aggregation-prone regions (V18-V24, and A30-V36), which are strongly hydrophobic, start to develop β-strands and form β-sheets early. These β-sheets then rearrange themselves so that a U-shape nucleus or a meta-stable oligomer with S-shape can form. The meta-stable oligomer eventually changes to a U-shape after which structural conversion to a protofilament structure occurs through monomeric addition templated by the U-shape nucleus.

### Statistics and validation

The preceding results describing energies, snapshots of structures, kinetic pathways and structural analyses are from 10 independent runs of 668 billion collisions (t*≈ 61,000). We performed another 100 independent runs more of 468 billion collisions (t*≈ 43,000) to get better statistics on the various conformations and to validate the generality of our suggested kinetic pathways. Besides the disordered, partially-ordered oligomers with U-shape or S-shape and mixed fibrillar structures containing more than two different fibril-like conformations as shown in S8C and S8H Fig, we observe nine fibrillar structures (S9 Fig), four fibril-like structures with the full S-shape conformations (S10 Fig) and three triangular fibril-like structures (S11 Fig). Among the nine fibrillar structures, we observe three trajectories along which there is a structural conversion from S-shape to U-shape (S12 Fig) and six trajectories in which there is U-shape nucleus formation and monomeric conversion process. We observed fewer ordered fibril structures and even fewer S-shape to U-shape pathways to reach the nice U-shape fibrillar structure than we expected. We see that some S-shape conformations further order without structural conversion to U-shape so that fibril-like structures with full S-shape conformations remain at the end of the simulation (S10 Fig), which means some S-shape conformations might be off-pathway. Actually simple structural conversion from S-shape to U-shape events and formation of partial U-shape are observed more frequently, but not all U-shape nuclei succeed at driving the system to form nice fibrillar structures. Hence many disordered or partially ordered oligomers are trapped and do not undergo further ordering. At present we do not know if this trapping and freezing of disordered structures is intrinsic to protein aggregation processes or is an artifact due to weaknesses of the coarse-grained model, since our force field has less detailed atomic movements and fluctuation preventing us from optimizing the fibril structures in atomic scale. We expect that our model’s ability to model on-pathway processes would be improved if we enhanced the stability of the U-shape nucleus by assigning known atomistic constraints such as the steric zipper interface by atomistic van der Waals interactions. Although we have not achieved unequivocal results for the fibrillar structures and kinetic pathways, we believe that our simulations provide a major leap forward in our ability to simulate the fibrillization process compared to other coarse-grained and all-atom simulations. In summary, by performing 100 independent runs, we reconfirm clearly two distinct kinetic on-pathways toward U-shape protofilaments.

## Discussion

The powerful combination of a four-sphere-per-residue protein model, PRIME20, and discontinuous molecular dynamics significantly facilitated the tracking of the aggregation process of peptide chains (Aβ17–42) that are longer than the 6 to 10 amino-acid peptides that have been considered usually in the past. Aβ17–42 is a good stand-in for its longer parent proteins Aβ1–42 and 1–40, because it contains the two hydrophobic stretches that dominate the aggregation and fibrillization of Aβ1–42 as well as the turn region. We consider this to be an important progress in the realistic modeling of Aβ oligomerization and fibrillization. Insights into the kinetic pathways and structural features for toxic oligomers that form spontaneously could be beneficial for the design of antibodies or small molecule inhibitors.

Our main focus has been to capture molecular-level insight on how random Aβ17–42 monomers turn into fibrillar structures through structural conversion via oligomers. We observe two different pathways during the structural change from disordered oligomers to ordered protofilament. The first pathway is one-by-one monomeric conversion templated by a U-shape nucleus; this is a fast process once the nucleus is formed. Although the monomeric conversion takes place within the oligomer, the nucleation and monomeric conversion share a common theme with other known mechanisms of fibril formation such as nucleated polymerization and the dock-lock mechanism by monomer addition.[48,49] The other pathway goes through a meta-stable oligomer with S-shape conformations due to the turn opportunities presented by the two flexible glycine residues (G37,G38); this adds considerably to the time it takes to convert to a U-shape nucleus and hence a protofilament. Experimental studies showed that designing a turn into the C-terminal region by mutation enhances the stability of the more toxic oligomers which are off-pathway species that are indeed detected in experiments.[14,17] Although the disc-shape pentamers containing S-shape monomers (from L17 to A42) observed by Smith and coworkers were considered by them to be on-pathway, the fact that their pentamer could only be converted to a fibril by raising the temperature suggests us that it was off-pathway.[8] Here we observe a meta-stable oligomer, which is very long lived but nevertheless on-pathway without changing temperature and which shares the S-shape that may be a characteristic of toxic oligomers as experiments suggested.[8] Teplow and coworkers also examined wild type Aβ42 oligomers and found that toxicity peaked at intermediate times.[14] Although they did not mention any evidence or possibility for a C-terminal turn, we speculate that this could be explained by the existence of a meta-stable on-pathway C-terminal turn at intermediate stage which caused toxicity but that this eventually converted to a less-toxic fibrillar structure.

The simulation temperatures of T* = 0.19 to 0.205, slightly below the fibrillization temperature were chosen to give us the best possible opportunity to watch the peptides evolve toward the lowest energy state. While this does introduce some artificiality by, in effect, smoothing the energy landscape, it has a number of advantages. The high entropic fluctuation that occurs in the reduced temperature range 0.19 ≤ T* ≤ 0.205 helps disordered oligomers to form in-register parallel β-sheets without getting trapped in meta-stable disordered states, as we have mentioned in a previous paper. Another advantage of simulating at a high temperature just below the transition temperature is that this condition slows down oligomerization as much as possible. It does this by preventing the trapping in large amorphous oligomeric states that usually accompanies rapid hydrophobic collapse. Instead it allows smaller oligomers to form alongside of the free monomers at an early simulation stage, making it easier for the oligomers to convert to ordered structures. A similar retardation of oligomerization could be achieved at lower protein concentration but this would have slowed down our simulations considerably. We do not have a satisfactory way at this time to relate the reduced temperature in our model to the real temperature. This is because the hydrogen bond energy which is used to scale the temperature is a potential of mean force due to the surrounding water rather than a direct interaction.

While we were able to construct fibrillized protofilaments successfully with our 8 chain systems after fairly long simulations runs, we also see a number of other very diverse structures including a triangle-shape β-sheets, β-helices and oligomers with β-strands arranged in a disordered fashion. This broad ensemble of structures may be a consequence of having a highly rugged energy landscape, which causes a variety of amorphous aggregates or polymorphic fibril conformations even in experiments, or it may partly depend on our having a coarse-grained force field like PRIME20 where the side-chain sizes and interactions are imprecise and not optimized for zipping up via van der Waals interactions between atoms. Nevertheless the impreciseness of the coarse-grained model is beneficial because it allows us to overcome the large energy barriers to achieving a fibrillar structure within our current capacity of computation.

## Methods

### PRIME20 model

We employ our new intermediate-resolution force field PRIME20 [38–40] in discontinuous molecular dynamics [50] simulations to study the aggregation of the Aβ17–42 peptide. PRIME20 is an extension of PRIME (Protein Intermediate-Resolution Model) and is designed to be applicable to all twenty amino acid residues.[38,51–53] The adequacy and efficiency of PRIME20 has been proven by applying to short peptide systems such as Aβ16–22, fragments of the prion proteins, the designed sequences of Lopez de la Paz et al, and the tau fragment (VQIVYK).[39,40,54,55] Aβ17–42 is modeled using the PRIME20 4-sphere-per-residue representation (backbone united atoms NH, CαH, and CO, and a single sphere side chain). The masses for the backbone united atom spheres are C_α_H(0.866), NH(0.999), CO(1.863), and for the side-chain united atom spheres are R(6.728), N(3.862), D(3.860), Q(4.795), E(4.793), H(5.394), K(4.865), S(2.064), T(2.997), A(1.000), I(3.799), L(3.799), M(4.998), F(6.061), Y(7.126), V(2.866) in mass units of CH_3_(15amu = 1.0). Each amino acid has a different set of geometric parameters including hard-sphere diameters and pair-interaction ranges which are given in our previous paper. The distances from the side-chain spheres to C_α_, NH, and CO united atoms are carefully designed to ensure that all amino acids remain in an L-isomer form during DMD simulations. We presented four Supporting Information Tables. The geometry distances for 20 amino acids are given in S1 Table, the minimum non-bonded distances between a side-chain sphere and other neighboring united backbone spheres called the squeeze parameters are given in S2 Table, the inner well diameters for double well potential are given S3 Table, and the pairwise interactions for double well potential are given in S4 Table. The outer well diameters are not included since they are given in the supplemental table 3 of the reference [38].

### Parallel preference constraints

In this simulation, we added two biases, parallel preference constraints and an enhanced D23-K28 salt-bridge interaction, because we were aiming to simulate the formation of parallel in-register U-shape protofilament which are experimentally observed for Aβ structure.[20,21] In order to reduce the potential complexity among polymorphic backbone orientation within β-sheets[6,42], we use the “parallel preference” set of distance cutoffs for backbone hydrogen bonds to enhance formation of parallel in-register β-sheets.[39] The parallel preference hydrogen-bond distance constraints between i-th donor residue and j-th acceptor residue are: N_i_-Cα_j_ (5.10Å), N_i_-N_j+1_ (4.54Å), C_j_-Cα_i_ (4.96Å), C_j_-C_i-1_ (4.58Å), the same as used on our simulations of the tau fragment [39]; it is slightly changed from the non-biased cutoff distances for directional hydrogen bonds used in our earlier papers: N_i_-Cα_j_ (5.00Å), N_i_-N_j+1_ (4.74Å), C_j_-Cα_i_ (4.86Å), C_j_-C_i-1_ (4.83Å).[40,55] These changes are obtained by measuring the distributions for these four distances in 620 NMR PDBs and decomposing them into distributions for parallel and anti-parallel β-sheets. This slight bias in our simulations, only 2–5% variation in cutoff distances, helps encourage the formation of parallel, as opposed to anti-parallel, pairs of β-strands. In our previous simulations of the tau fragment (VQIVYK), the implementation of parallel preference constraints converted the final configuration from β-sheets with a random mixture of parallel and anti-parallel β-strands in the original H-bond constraints to nearly perfect parallel β-sheets as had been seen experimentally.[39] Hence the parallel preference directional hydrogen bonds can assist in promoting in-register β-sheets and suppressing mixed β-sheets with parallel and anti-parallel pairs of strands. This is a reasonable approximation since the difference between the formation energy of a parallel β-sheet and an anti-parallel β-sheet in all atom simulations and coarse-grained models is very small. This slight bias greatly enhances the possibility to form perfect Aβ fibril structures with parallel in-register U-shape β-sheets. Although we obtain nice results for Aβ17–42 peptides and tau fragments (VQIVYK) systems, this bias is obviously not applicable to other systems such as Aβ16–22 peptides which are known to form highly anti-parallel β-sheet.[40] If we apply parallel preference constraints to Aβ16–22 peptides, we observe mixed β-sheets having almost half parallel and half anti-parallel pairs of strands.

### Enhanced salt-bridge interaction

We also use an enhanced salt-bridge interaction between K28 and D23 residues. This salt-bridge is critical to fibril formation in Aβ. Forming and burying the salt-bridge inside a protofilament is believed to generate a high energy barrier and hence is a rate-limiting step.[33,56] Thus the enhancing the possibility that a salt bridge will form in experiments, for example by making a Lactam bond between D23 and K28, can significantly increase the fibrillation rate.[57] It is interesting to note that the salt-bridge is not as highly populated in quiescent experiments as it is in agitated fibrillation experiment.[43] Hence enhancing the possibility of salt bridge formation in simulation is quite reasonable. It helps the disordered oligomers to move easily on the free energy surface toward the expected U-shape protofilament structure; essentially reducing the ruggedness of the energy landscape. We increase the pair interaction value from its original value in PRIME20 of 0.136ε_HB_ to 0.4ε_HB,_ which is almost twice the strength of the strong hydrophobic side-chain interaction (ε_F-F_ = 0.205ε_HB_).

For comparison, we present the results from 10 more independent simulations with non-enhanced salt-bridge interactions (ε_KD_ = 0.136ε_HB_) performed for 468 billion collisions. The final structures are shown in S13 Fig. Partial U-shape structures are observed in S13A and S13G Fig but they are mixed fibrillar structures consisting of more than two different fibril-like conformations. S13A Fig contains triangular shape and U-shape conformations. S13G Fig contains two U-shape conformations oriented in different directions. The average numbers of salt-bridge interactions per structure during the last 50 billion collisions are 0.13±0.24 for intramolecular and 0.16±0.29 for intermolecular interactions, while simulations with the enhanced salt-bridge interaction give numbers of 1.7±1.4 for intramolecular and 2.2±2.0 for intermolecular interactions at the same time and the same temperature. Therefore we can see that the number of salt-bridge interactions is greatly increased when using the enhanced salt-bridge interaction condition. Roughly speaking, S13 Fig looks similar to the other disordered structures, such as S2 Fig, but the nice fibrillar structures are far less accessible since the probability of salt-bridge interactions is very low.

While these two biases clearly enhance the likelihood of fibrillization and allow us to skip some of the meta-stable structures along the way, they also could alter the oligomerization mechanism. However we think the important mechanisms or structural insights for oligomerization and fibril formation are being captured. Actually these nice well-organized fibrillar structures could not be obtained spontaneously without those two biases. And if we could not obtain any nice fibrillar structures (which we know do form), we would not know whether our intermediate structures in simulations are real oligomers observable in experiments or artifacts of simulations (trapped meta-stable oligomers in highly rugged energy landscape seen in the most simulations). So the biased simulations may look ad-hoc, but they are necessary if we want to examine spontaneous fibrillation given our present computational limitations.

### Double well potential

In addition to the above two biases (compared to the original PRIME20) mentioned above, we add another improvement to the PRIME20 model. Instead of the standard single well potential we use double well potentials for every pair interaction between side-chain spheres. In our previous studies [39,40] interactions between side-chain spheres were modeled using a single well potential. The well diameters were evaluated from the pairwise distance distributions between the side-chain spheres over 711 PDBs. Each pairwise interaction was included in the pairwise distribution when over half of the distances between heavy atoms on different side-chains were less than 5.5Å cut-off (called 5.5Å heavy atom criteria), as is explained in detail in the previous work.[38] In this paper we use a double well potential; the previous single well is replaced by two wells—an inner deep well and an outer shallow well. We determine the inner deep well diameter using a 4.5Å heavy atom criteria and the outer shallow well diameter using a 5.5Å heavy atom criteria. The depth of the inner deep well is taken to be 1.3ε(*ij*) and the depth of the outer shallow well depth is taken to be 0.7ε(*ij*) where ε(*ij)* are the well depths (pair interaction strength) from the original 19 parameter PRIME20 force field [38], which were estimated by the perceptron learning algorithm. We observed very similar results in Aβ16–22 simulations for the single well potentials and the double well potentials but felt that a more detailed force field might be necessary for longer chain systems like the Aβ17–42 peptide.

## Supporting Information

S1 Fig Size dependence of Aβ17–42 peptides. Representative or selective structures of Aβ17–42 peptides at T* = 0.20 with (A) 1, (B) 2, (C) 4, (D) 5, (E) 6 peptide chains. Simulations have been performed on 1mM concentration for 468 billion collisions.
(TIF)

S2 Fig The other seven structures. Seven final structures for 8 Aβ17–42 peptides among 10 independent runs at T* = 0.20. Structures for the (A) 1^st^, (B) 2^nd^, (C) 4^th^, (D) 6^th^, (E) 7^th^, (F) 8^th^, (G) 9th runs after 668 billion collisions (t* ≈ 61,000).
(TIF)

S3 Fig Glycine residues in the structures. Location of the glycine residues in the structures shown in Fig 1 for (A) (B) the 3^rd^ run, (C) (D) the 5^th^ run, (E) (F) the 10^th^ run. The GLY residues are colored G25(blue), G29(yellow), G33(red), G37(orange) and G38(orange) to show how the glycine residues contribute to the turns in the β-strands. For the 3^rd^ run in (A) and (B), two chains (cyan) are anti-parallel to the other six chains (gray) but have the same turning residues G25 and G33, so that a triangular-shape is formed.
(TIF)

S4 Fig Salt-bridge and hydrophobic interactions. (A) Structure at 568 billion collisions (t*≈52,000) for the 5^th^ run. (B)(C) The fibril axis view with ribbon diagram or with side-chain spheres. (D)-(K) Fibril axis views for each chain showing side-chain spheres; F19(purple), D23(red), K28(cyan), I32(green) and L34(pink sphere). Only figures (I) and (J) show the salt-bridge pairs (D23-K28) and hydrophobic interactions between I32, L34 and F19.
(TIF)

S5 Fig Structures in very early stage. Early snapshots for the 5th run and the 10th run. Snapshots are taken for the 5th run at (A) t* = 306, (B) 605 and for the 10th run at (C) t* = 306, (D) 605, (E) 1806. Snapshots at t* = 306 and 605 are within the slow cooling stage from T* = 0.50 to T* = 0.20 over the course of the first 8 billion collisions (t* = 788) so that disordered monomers or dimers are prevail.
(TIF)

S6 Fig Stability measured by all-atom simulations. The observables measuring the stability for seven structures (C,D,E,F,G,H,I) of Fig 4C–4I and seven structures (C,D,E,F,G,H,I) of Fig 5C–5I by all-atom MD simulations. System energy for (A) the 5th run and (B) the 10th run includes internal, electrostatic, van der Waals and solvation (GB, SA) energies. Binding energy for (C) the 5th run and (D) the 10th run is estimated by subtracting the effective energy of the separate monomers from the system energy. Backbone atoms’ RMSF(root mean square fluctuation) are presented for (E) the 5th run and (F) for the 10th run.
(TIF)

S7 Fig Structures at T* = 0.198. Ten final structures for 8 Aβ17–42 peptides from 10 independent runs at T* = 0.198. Structures are taken after 668 billion collisions (t* ≈ 61000).
(TIF)

S8 Fig Structures at T* = 0.202. Ten final structures for 8 Aβ17–42 peptides from 10 independent runs at T* = 0.202. Structures are taken after 668 billion collisions (t* ≈ 61000).
(TIF)

S9 Fig Nice fibrils. Nine structures showing nice fibrillar structures from 100 independent runs simulated for relatively short times, 468 billion collisions (t*≈ 43,000).
(TIF)

S10 Fig Full S-shape. Four fibril-like structures with full S-shape conformations. Snapshots are taken at 468 billion collisions (t*≈ 43,000).
(TIF)

S11 Fig Triangular-shape. Three triangular-shape fibril-like structures. Snapshots are taken at 468 billion collisions (t*≈ 43,000).
(TIF)

S12 Fig From S-shape to U-shape. Three trajectories showing structural conversion from S-shape to U-shape conformation which are simulated for 468 billion collisions (t*≈ 43,000).
(TIF)

S13 Fig Structures without enhanced salt-bridge interactions. By using the non-enhanced salt-bridge interactions (ε_KD_ = 0.136ε_HB_), ten final structures for 8 Aβ17–42 peptides from 10 independent runs at T* = 0.20 are found after 368 billion collisions.
(TIF)

S1 Table Geometry distances for 20 amino acids. Parameters for covalent bond distance of Cα to side-chain sphere (DRCα), pseudo-bond distances of NH united sphere to side-chain sphere (DRNH) and of CO united sphere to side-chain sphere (DRCO).
(DOC)

S2 Table Squeeze parameters. The minimum distances between neighboring spheres not having covalent or pseudo-bonds. Distances of a side-chain sphere of i-th residue to a Cα sphere of i-1th residue (R(i) to Cα(i-1)), of a side-chain sphere of i-th residue to a CO sphere of i-1th residue (R(i) to CO(i-1)), of a side-chain sphere of i-th residue to a NH sphere of i+1 sphere (R(i) to NH(i+1)), of a side-chain sphere of i-th residue to a Cα sphere of i+1th residue (R(i) to Cα(i+1)), of a CO sphere of i-1th residue to a side-chain sphere of i+1th residue (CO(i-1) to R(i+1)).
(DOC)

S3 Table Inner well diameters. Inner well diameters (Å) between side-chain centroids which are estimated from 711 PDBs with heavy atom distance cutoff 4.5Å. For outer well diameters, see the supplemental table 3 of the reference [38].
(DOC)

S4 Table 19 independent energy parameters for double well potentials. We multiply the original 19 parameters (Table III in reference [38]) by 1.3 to get the energy for the inner deep well and by 0.7 to get the energy for the outer shallow well. The salt-bridge between K and D is enhanced as -0.4×1.3 for the inner deep well and -0.4×0.7 for the outer shallow well. For detail classifications of parameters, please see reference [38].
(DOC)

S1 Video Structural conversion of the 5^th^ run. The 5^th^ run trajectory of eight Aβ17–42 peptides by DMD till t* = 11,063 (121 billion collisions). Conformational conversion occurs by one by one templating of monomers on the U-shape nucleus.
(MP4)

S2 Video Structural conversion of the 10^th^ run. The 10^th^ run trajectory of eight Aβ17–42 peptides by DMD till t* = 37,943 (413 billion collisions). An oligomer with partial S-shape chains is meta-stable and remains for a long time before finally converting to U-shape structure.
(MP4)

S3 Video Constant structural switching. Two directional views at late time for the 5^th^ run trajectory from t* = 33,500 to 42,736. Intra-chain interactions forming the U-shape β-sheet constantly change to inter-chain interactions and vice versa.
(MP4)
