# Supplementary figures and images for: Structural Conversion of Aβ17–42 Peptides from Disordered Oligomers to U-Shape Protofilaments via Multiple Kinetic Pathways
Source: PLoS Comput Biol. 2015 May 8;11(5):e1004258. doi: 10.1371/journal.pcbi.1004258 (PMC4425657; doi:10.1371/journal.pcbi.1004258)

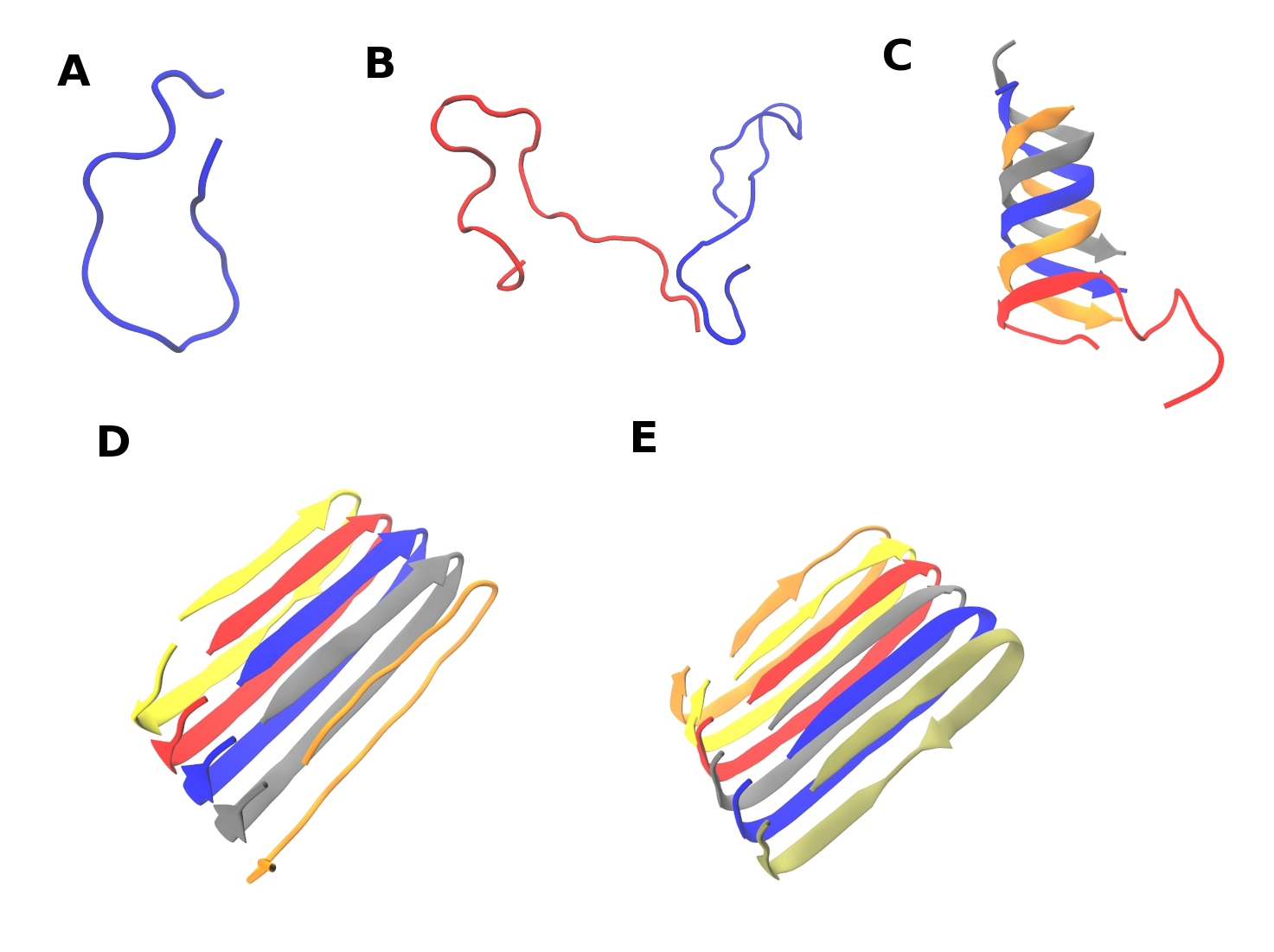

Supplement: S1 Fig — Representative or selective structures of Aβ17–42 peptides at T* = 0.20 with (A) 1, (B) 2, (C) 4, (D) 5, (E) 6 peptide chains. Simulations have been performed on 1mM concentration for 468 billion collisions. (TIF) [file pcbi.1004258.s001.tif]

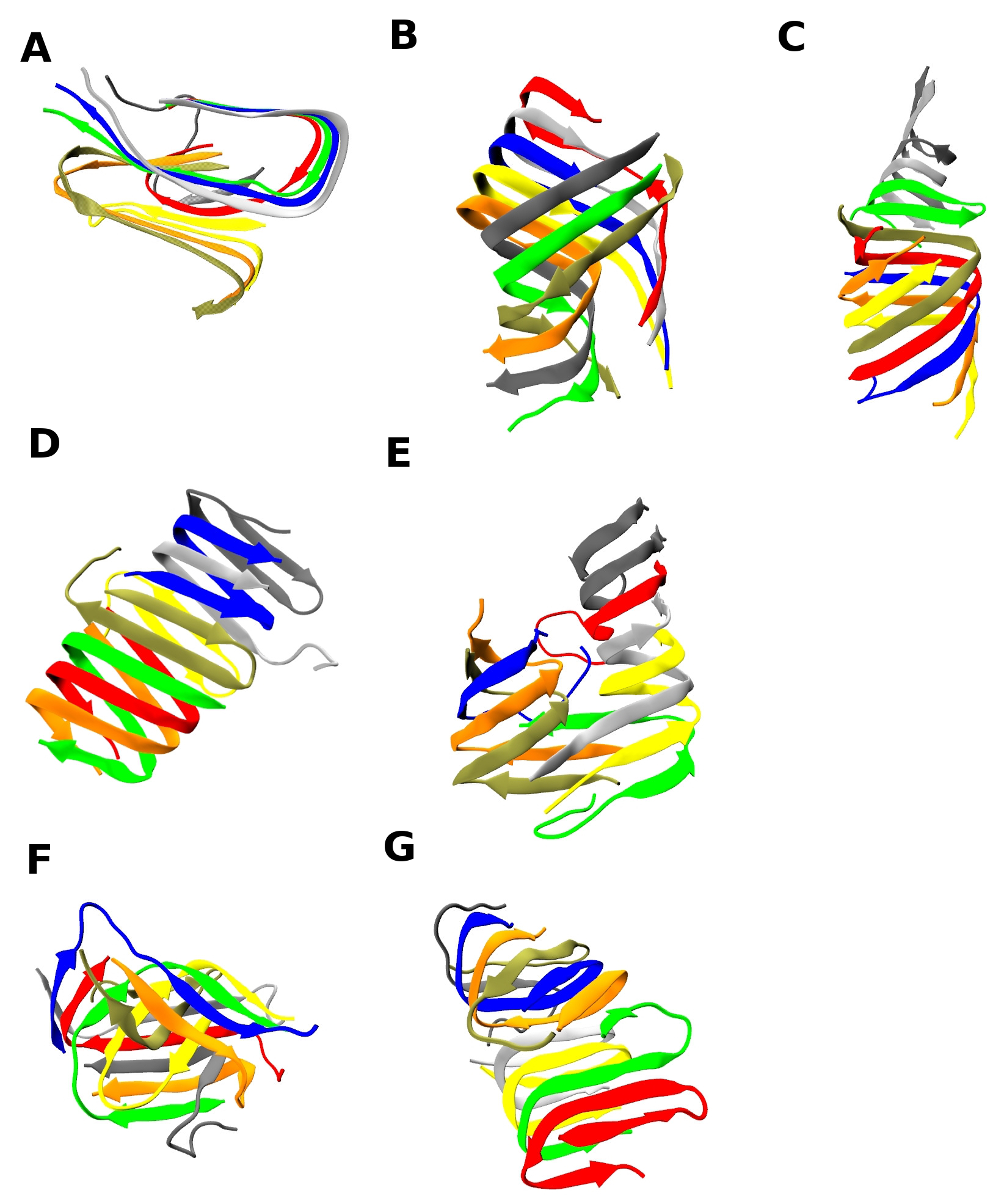

Supplement: S2 Fig — Seven final structures for 8 Aβ17–42 peptides among 10 independent runs at T* = 0.20. Structures for the (A) 1st, (B) 2nd, (C) 4th, (D) 6th, (E) 7th, (F) 8th, (G) 9th runs after 668 billion collisions (t* ≈ 61,000). (TIF) [file pcbi.1004258.s002.tif]

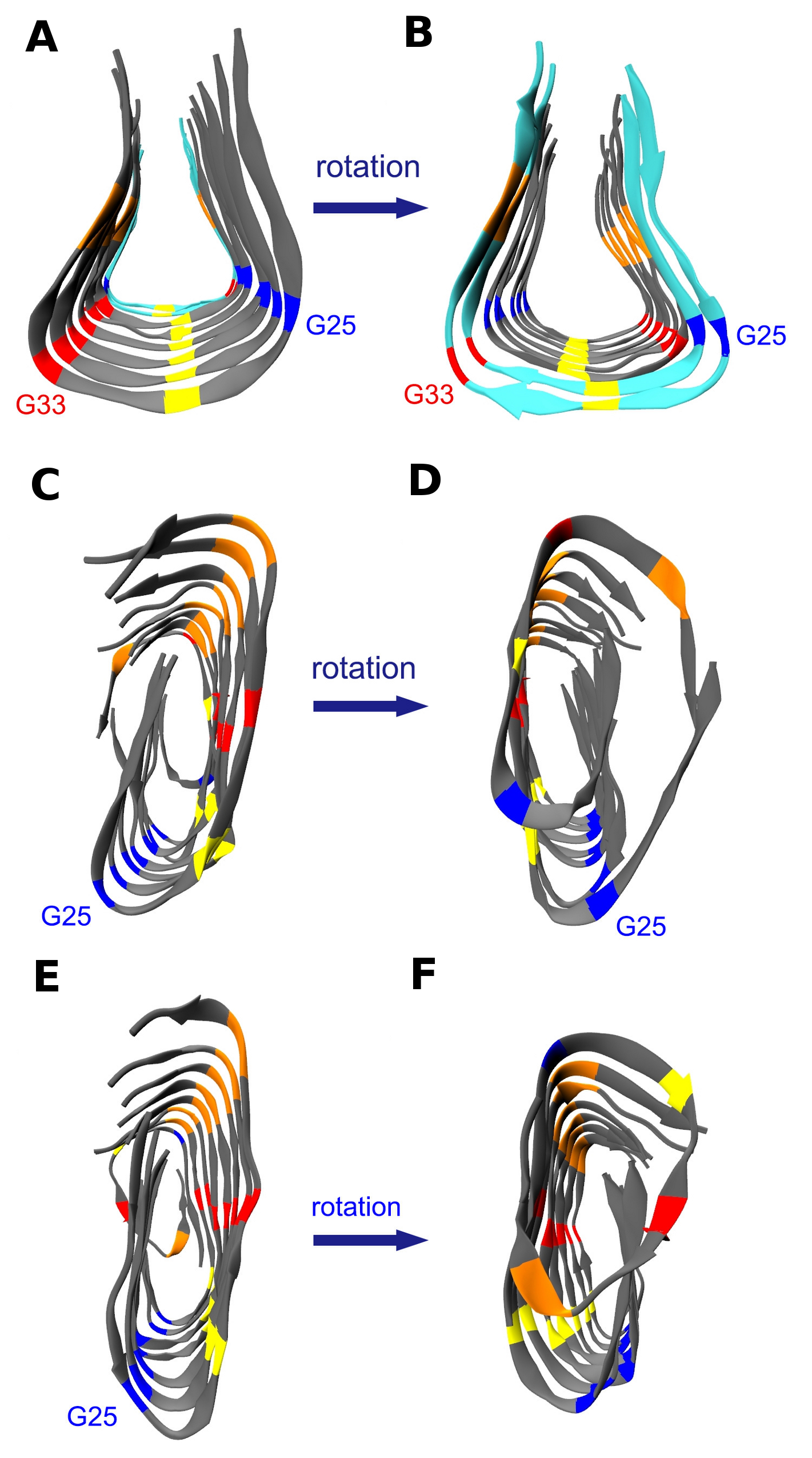

Supplement: S3 Fig — Location of the glycine residues in the structures shown in Fig 1 for (A) (B) the 3rd run, (C) (D) the 5th run, (E) (F) the 10th run. The GLY residues are colored G25(blue), G29(yellow), G33(red), G37(orange) and G38(orange) to show how the glycine residues contribute to the turns in the β-strands. For the 3rd run in (A) and (B), two chains (cyan) are anti-parallel to the other six chains (gray) but have the same turning residues G25 and G33, so that a triangular-shape is formed. (TIF) [file pcbi.1004258.s003.tif]

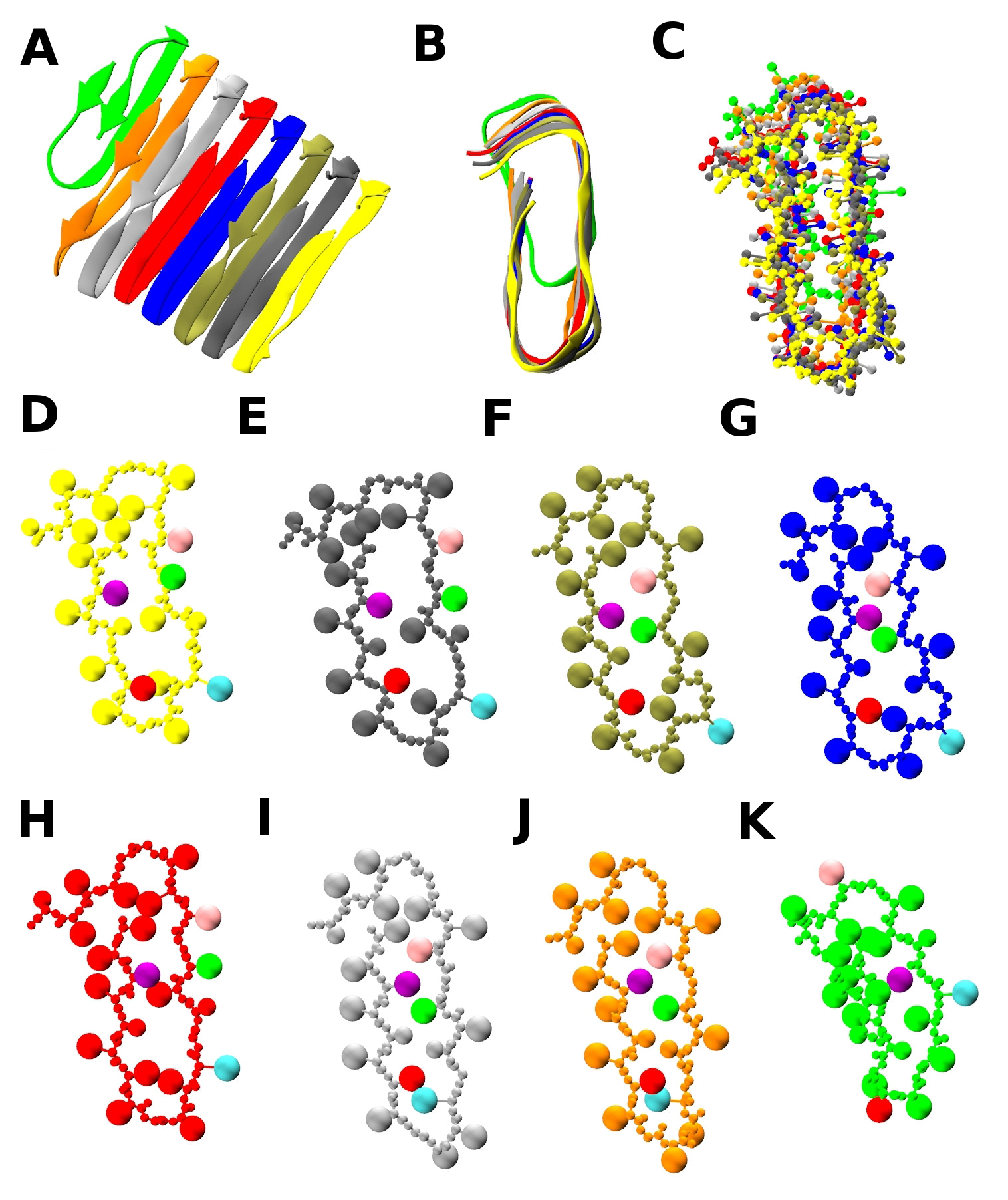

Supplement: S4 Fig — (A) Structure at 568 billion collisions (t*≈52,000) for the 5th run. (B)(C) The fibril axis view with ribbon diagram or with side-chain spheres. (D)-(K) Fibril axis views for each chain showing side-chain spheres; F19(purple), D23(red), K28(cyan), I32(green) and L34(pink sphere). Only figures (I) and (J) show the salt-bridge pairs (D23-K28) and hydrophobic interactions between I32, L34 and F19. (TIF) [file pcbi.1004258.s004.tif]

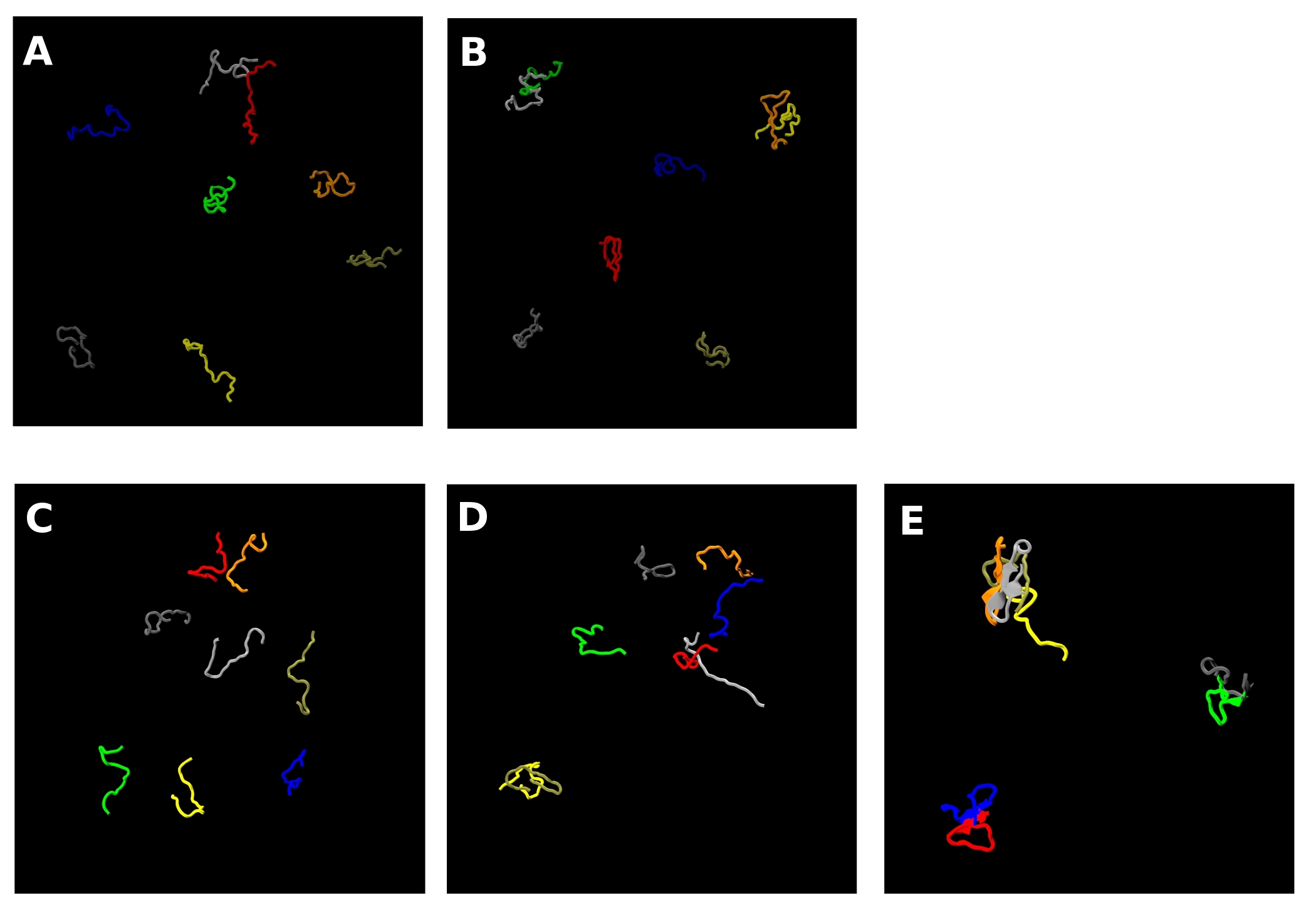

Supplement: S5 Fig — Early snapshots for the 5th run and the 10th run. Snapshots are taken for the 5th run at (A) t* = 306, (B) 605 and for the 10th run at (C) t* = 306, (D) 605, (E) 1806. Snapshots at t* = 306 and 605 are within the slow cooling stage from T* = 0.50 to T* = 0.20 over the course of the first 8 billion collisions (t* = 788) so that disordered monomers or dimers are prevail. (TIF) [file pcbi.1004258.s005.tif]

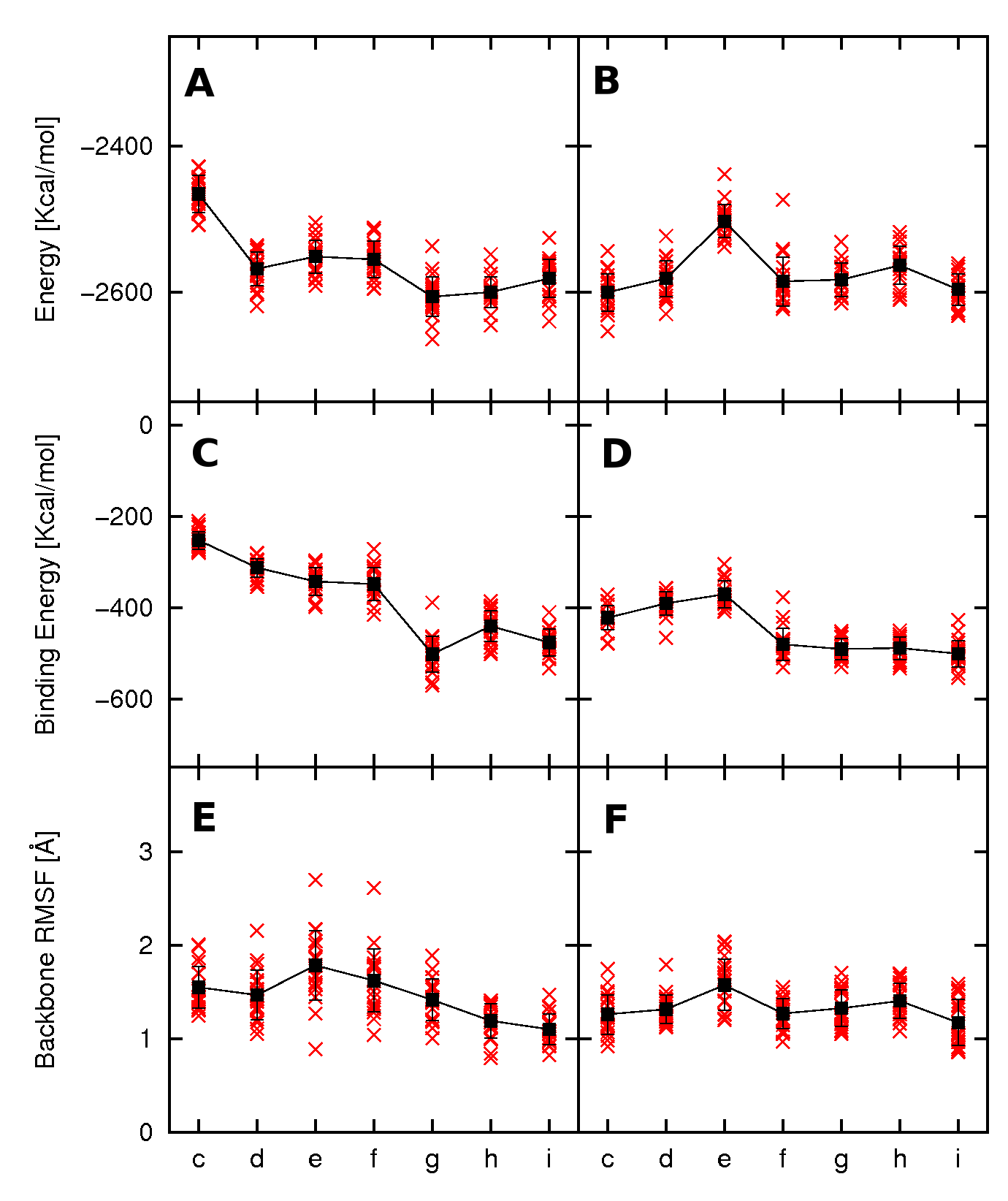

Supplement: S6 Fig — The observables measuring the stability for seven structures (C,D,E,F,G,H,I) of Fig 4C–4I and seven structures (C,D,E,F,G,H,I) of Fig 5C–5I by all-atom MD simulations. System energy for (A) the 5th run and (B) the 10th run includes internal, electrostatic, van der Waals and solvation (GB, SA) energies. Binding energy for (C) the 5th run and (D) the 10th run is estimated by subtracting the effective energy of the separate monomers from the system energy. Backbone atoms’ RMSF(root mean square fluctuation) are presented for (E) the 5th run and (F) for the 10th run. (TIF) [file pcbi.1004258.s006.tif]

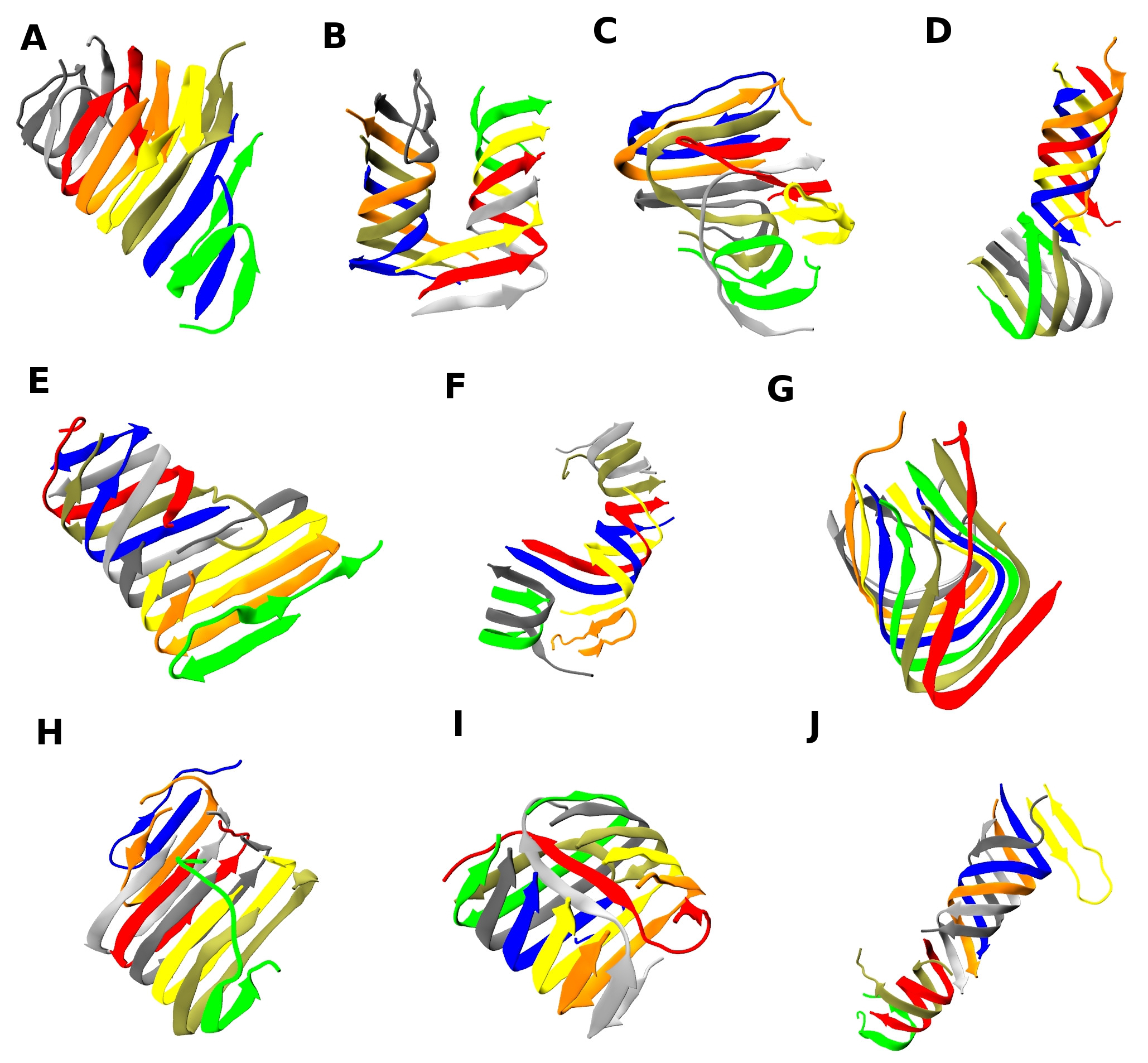

Supplement: S7 Fig — Ten final structures for 8 Aβ17–42 peptides from 10 independent runs at T* = 0.198. Structures are taken after 668 billion collisions (t* ≈ 61000). (TIF) [file pcbi.1004258.s007.tif]

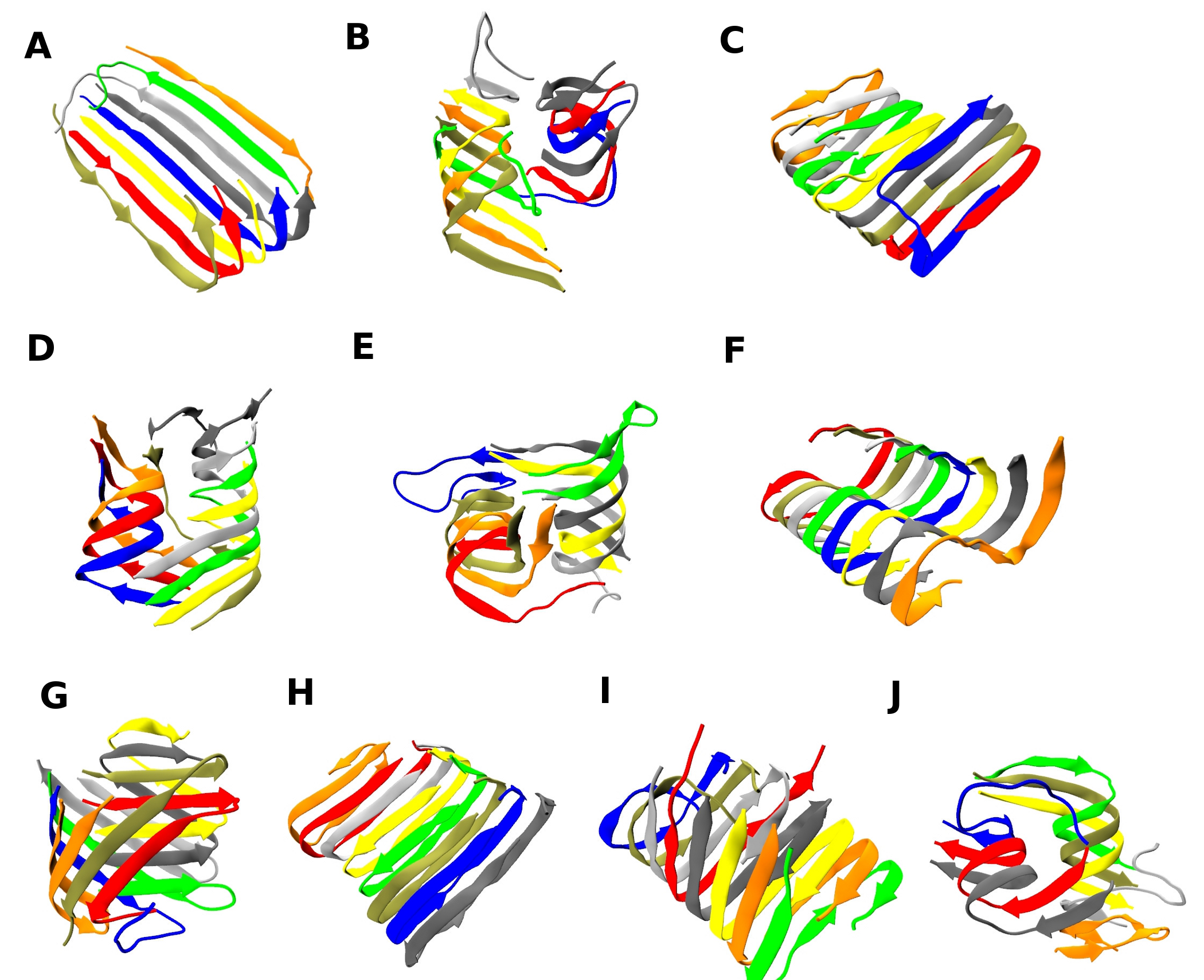

Supplement: S8 Fig — Ten final structures for 8 Aβ17–42 peptides from 10 independent runs at T* = 0.202. Structures are taken after 668 billion collisions (t* ≈ 61000). (TIF) [file pcbi.1004258.s008.tif]

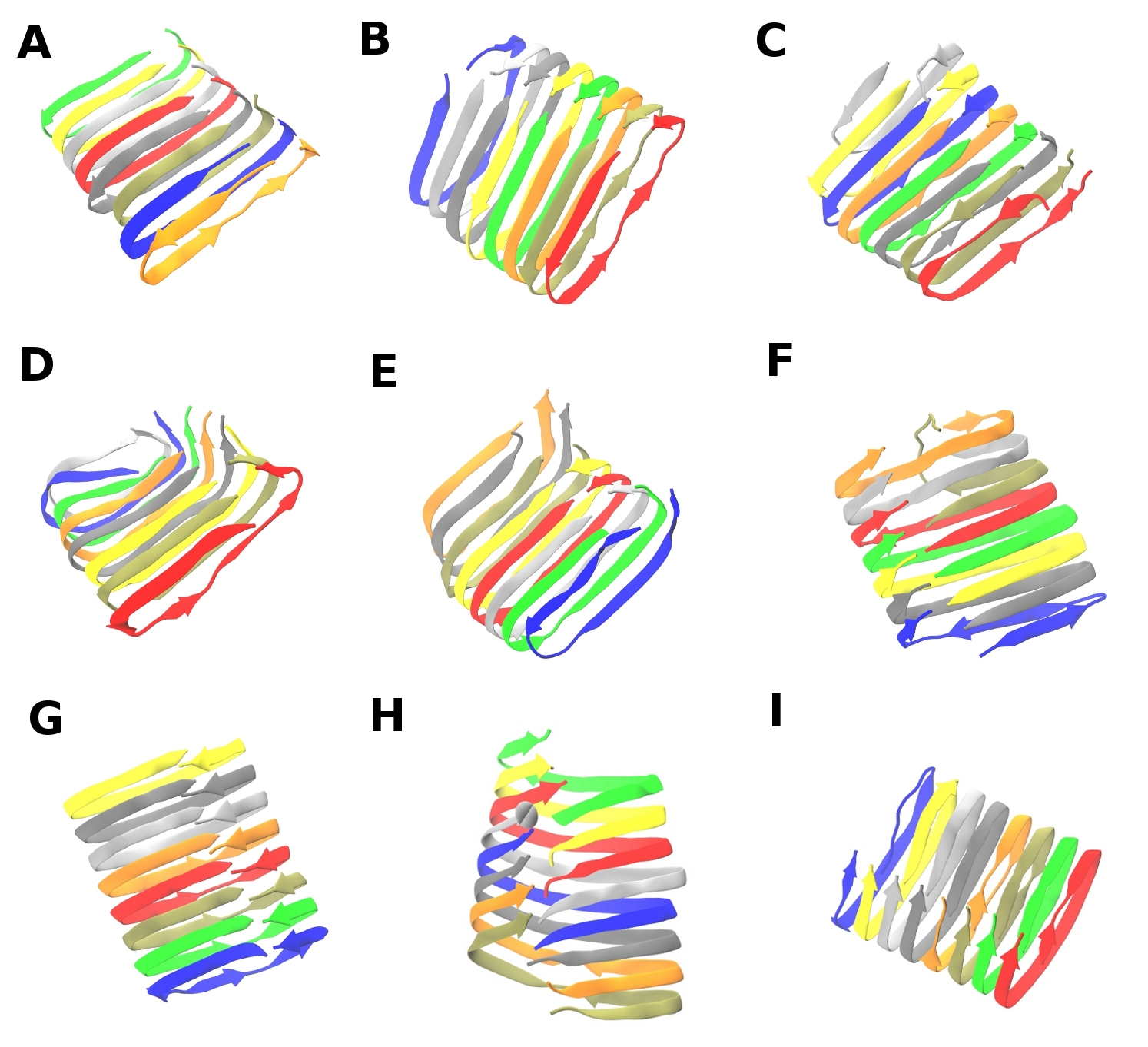

Supplement: S9 Fig — Nine structures showing nice fibrillar structures from 100 independent runs simulated for relatively short times, 468 billion collisions (t*≈ 43,000). (TIF) [file pcbi.1004258.s009.tif]

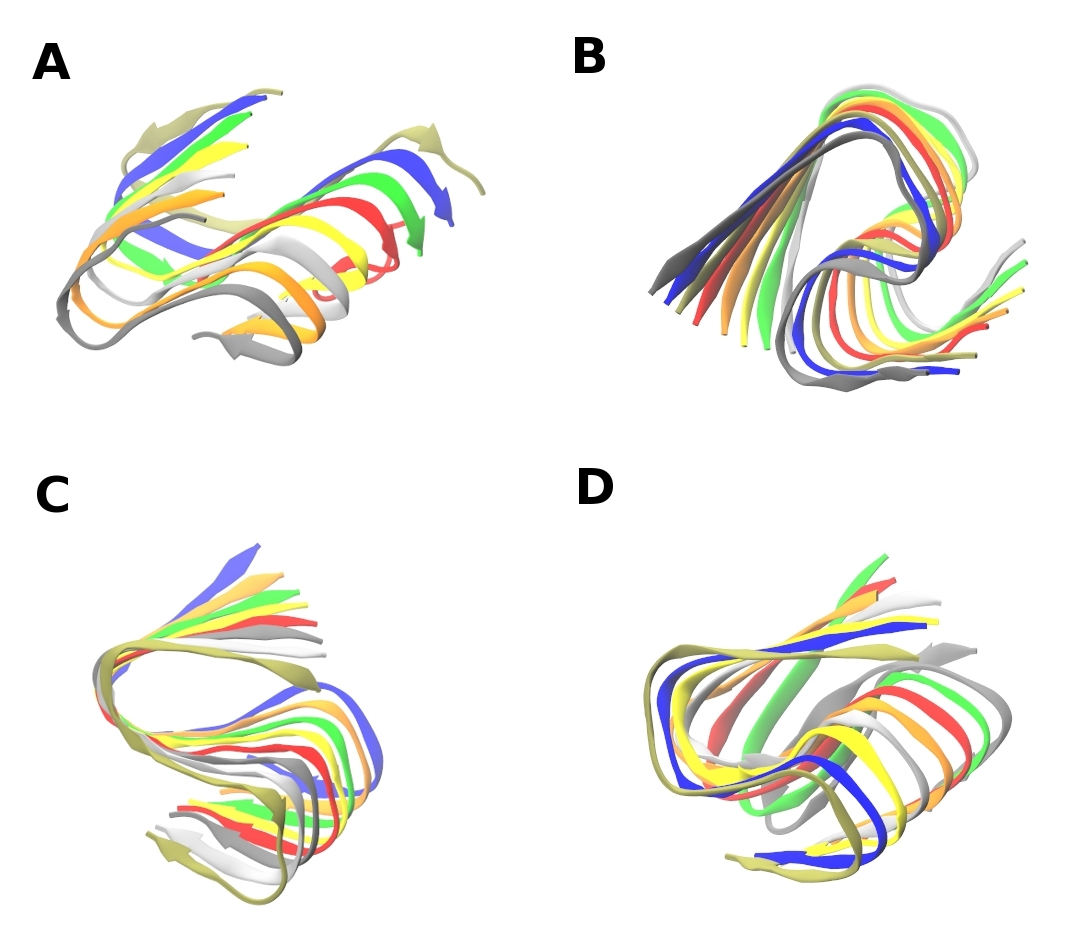

Supplement: S10 Fig — Four fibril-like structures with full S-shape conformations. Snapshots are taken at 468 billion collisions (t*≈ 43,000). (TIF) [file pcbi.1004258.s010.tif]

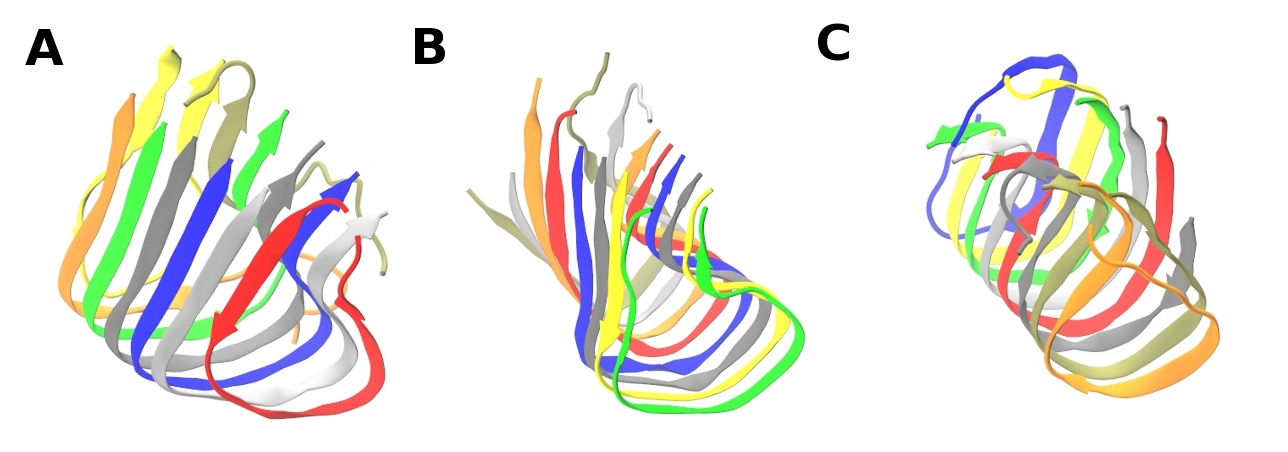

Supplement: S11 Fig — Three triangular-shape fibril-like structures. Snapshots are taken at 468 billion collisions (t*≈ 43,000). (TIF) [file pcbi.1004258.s011.tif]

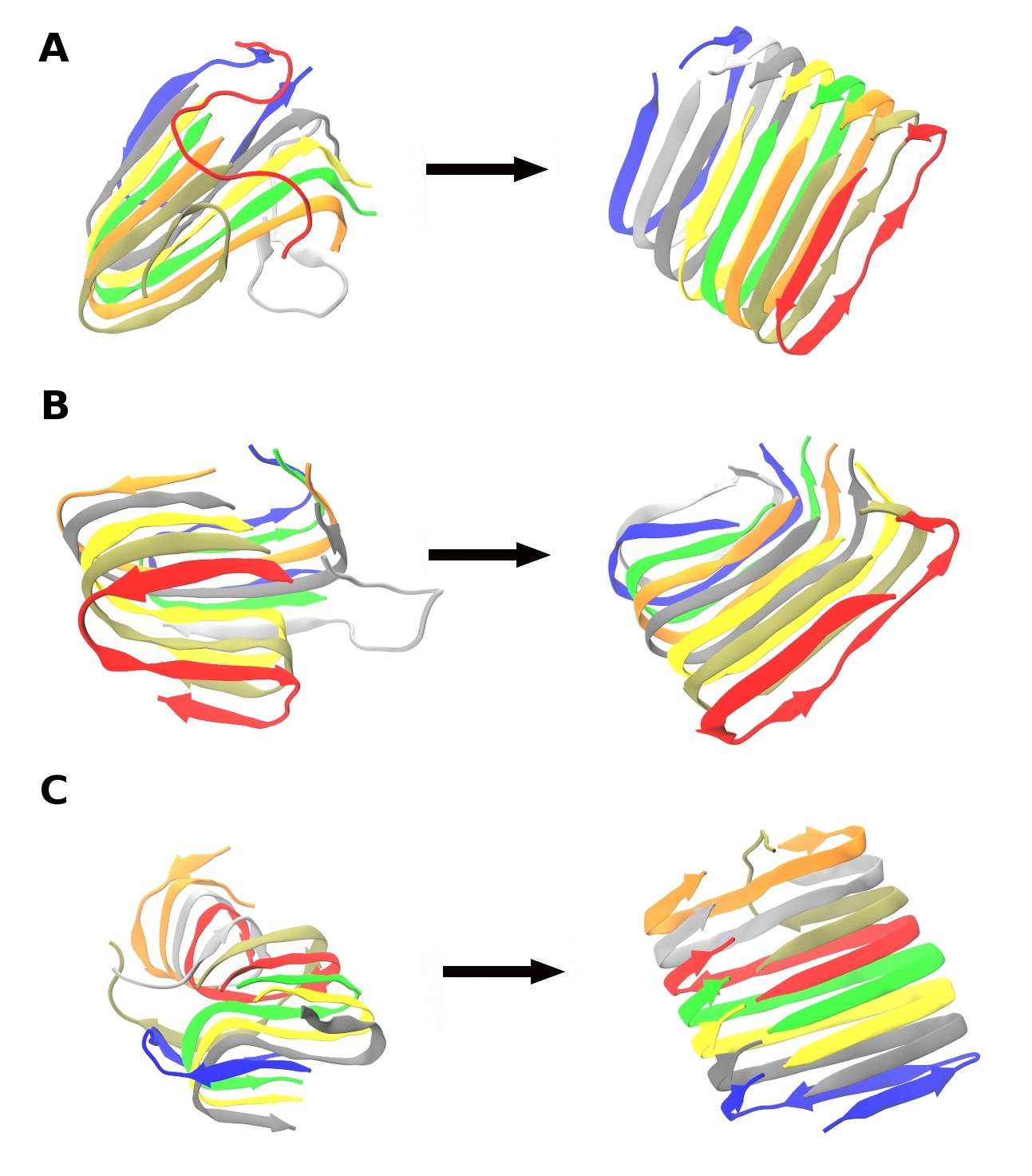

Supplement: S12 Fig — Three trajectories showing structural conversion from S-shape to U-shape conformation which are simulated for 468 billion collisions (t*≈ 43,000). (TIF) [file pcbi.1004258.s012.tif]

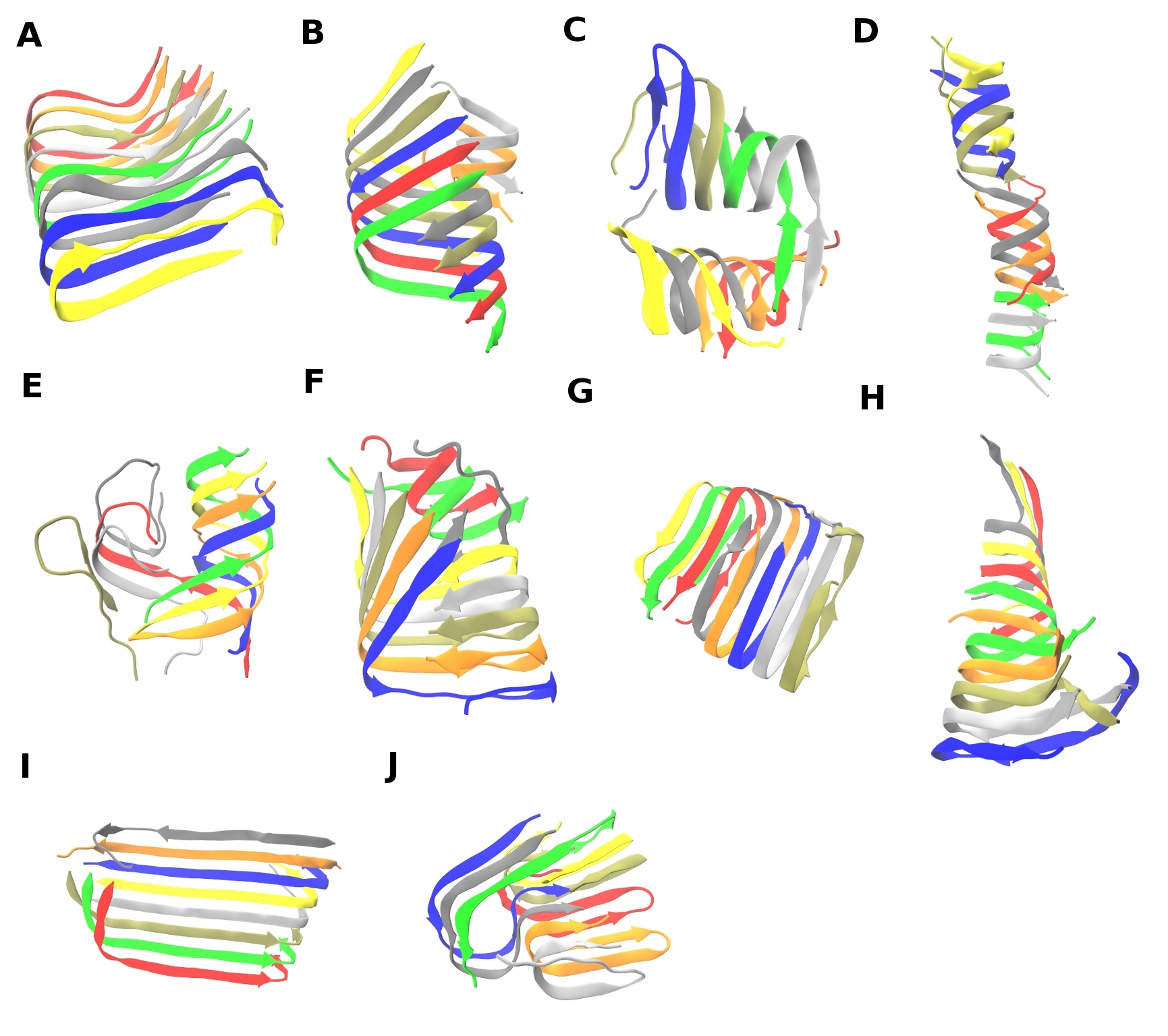

Supplement: S13 Fig — By using the non-enhanced salt-bridge interactions (εKD = 0.136εHB), ten final structures for 8 Aβ17–42 peptides from 10 independent runs at T* = 0.20 are found after 368 billion collisions. (TIF) [file pcbi.1004258.s013.tif]
