# Supplementary material for: Structural Conversion of Aβ17–42 Peptides from Disordered Oligomers to U-Shape Protofilaments via Multiple Kinetic Pathways
Source: PLoS Comput Biol. 2015 May 8;11(5):e1004258. doi: 10.1371/journal.pcbi.1004258 (PMC4425657; doi:10.1371/journal.pcbi.1004258)
Supplement: S1 Table — Parameters for covalent bond distance of Cα to side-chain sphere (DRCα), pseudo-bond distances of NH united sphere to side-chain sphere (DRNH) and of CO united sphere to side-chain sphere (DRCO). (DOC) [file pcbi.1004258.s014.doc]

| **Amino Acids** | **DRC_α_(Å)** | **DRNH(Å)** | **DRCO(Å)** |
| --- | --- | --- | --- |
| **R** | **4.200** | **4.500** | **4.800** |
| **N** | **2.510** | **3.050** | **3.350** |
| **D** | **2.500** | **3.100** | **3.250** |
| **Q** | **3.300** | **3.750** | **4.000** |
| **E** | **3.180** | **3.780** | **3.930** |
| **H** | **3.160** | **3.450** | **3.830** |
| **K** | **3.550** | **4.050** | **4.250** |
| **P** | **1.926** | **1.851** | **2.995** |
| **S** | **1.967** | **2.650** | **2.800** |
| **T** | **1.981** | **2.650** | **2.900** |
| **A** | **1.600** | **2.500** | **2.560** |
| **C** | **2.350** | **2.800** | **3.100** |
| **I** | **2.400** | **3.050** | **3.300** |
| **L** | **2.625** | **3.290** | **3.500** |
| **M** | **3.400** | **3.800** | **4.050** |
| **F** | **3.425** | **3.650** | **4.050** |
| **W** | **3.881** | **4.100** | **4.350** |
| **Y** | **3.843** | **4.050** | **4.300** |
| **V** | **2.002** | **2.775** | **2.959** |

**S1 Table. Geometry distances for 20 amino acids.** Parameters for covalent bond distance of Cα to side-chain sphere (DRCα), pseudo-bond distances of NH united sphere to side-chain sphere (DRNH) and of CO united sphere to side-chain sphere (DRCO).
