# Supplementary material for: Structural Conversion of Aβ17–42 Peptides from Disordered Oligomers to U-Shape Protofilaments via Multiple Kinetic Pathways
Source: PLoS Comput Biol. 2015 May 8;11(5):e1004258. doi: 10.1371/journal.pcbi.1004258 (PMC4425657; doi:10.1371/journal.pcbi.1004258)
Supplement: S2 Table — The minimum distances between neighboring spheres not having covalent or pseudo-bonds. Distances of a side-chain sphere of i-th residue to a Cα sphere of i-1th residue (R(i) to Cα(i-1)), of a side-chain sphere of i-th residue to a CO sphere of i-1th residue (R(i) to CO(i-1)), of a side-chain sphere of i-th residue to a NH sphere of i+1 sphere (R(i) to NH(i+1)), of a side-chain sphere of i-th residue to a Cα sphere of i+1th residue (R(i) to Cα(i+1)), of a CO sphere of i-1th residue to a side-chain sphere of i+1th residue (CO(i-1) to R(i+1)). (DOC) [file pcbi.1004258.s015.doc]

| Amino Acids | R(i) to Cα(i-1) | R(i) to CO(i-1) | R(i) to NH(i+1) | R(i) to Cα(i+1) | CO(i-1) to R(i+1) |
| --- | --- | --- | --- | --- | --- |
| R | 5.703 | 4.827 | 4.651 | 5.535 | 4.978 |
| N | 4.633 | 3.607 | 3.565 | 4.680 | 4.791 |
| D | 4.785 | 3.751 | 3.435 | 4.558 | 4.860 |
| Q | 5.134 | 4.139 | 3.996 | 5.062 | 5.000 |
| E | 5.162 | 4.175 | 3.997 | 5.074 | 4.996 |
| H | 4.766 | 3.886 | 3.838 | 4.790 | 4.945 |
| K | 5.323 | 4.384 | 4.191 | 5.163 | 4.974 |
| P | 3.884 | 3.133 | 3.298 | 4.665 | 4.773 |
| S | 4.507 | 3.331 | 3.128 | 4.380 | 4.944 |
| T | 4.617 | 3.447 | 3.290 | 4.573 | 5.007 |
| A | 4.598 | 3.312 | 3.000 | 4.353 | 4.997 |
| C | 4.560 | 3.516 | 3.350 | 4.501 | 4.913 |
| I | 4.867 | 3.740 | 3.626 | 4.867 | 4.994 |
| L | 4.936 | 3.918 | 3.724 | 4.863 | 5.001 |
| M | 5.206 | 4.205 | 4.032 | 5.067 | 5.017 |
| F | 4.780 | 3.991 | 3.973 | 4.827 | 5.040 |
| W | 5.180 | 4.460 | 4.187 | 4.963 | 4.986 |
| Y | 4.898 | 4.208 | 4.246 | 4.978 | 5.042 |
| V | 4.754 | 3.570 | 3.378 | 4.635 | 5.002 |

**S2 Table. Squeeze parameters.** The minimum distances between neighboring spheres not having covalent or pseudo-bonds. Distances of a side-chain sphere of i-th residue to a Cα sphere of i-1th residue (R(i) to Cα(i-1)), of a side-chain sphere of i-th residue to a CO sphere of i-1th residue (R(i) to CO(i-1)), of a side-chain sphere of i-th residue to a NH sphere of i+1 sphere (R(i) to NH(i+1)), of a side-chain sphere of i-th residue to a Cα sphere of i+1th residue (R(i) to Cα(i+1)), of a CO sphere of i-1th residue to a side-chain sphere of i+1th residue (CO(i-1) to R(i+1)).
