# Supplementary material for: Structural Conversion of Aβ17–42 Peptides from Disordered Oligomers to U-Shape Protofilaments via Multiple Kinetic Pathways
Source: PLoS Comput Biol. 2015 May 8;11(5):e1004258. doi: 10.1371/journal.pcbi.1004258 (PMC4425657; doi:10.1371/journal.pcbi.1004258)
Supplement: S3 Table — Inner well diameters (Å) between side-chain centroids which are estimated from 711 PDBs with heavy atom distance cutoff 4.5Å. For outer well diameters, see the supplemental table 3 of the reference [38]. (DOC) [file pcbi.1004258.s016.doc]

|  | **A** | **V** | **P** | **T** | **S** | **N** | **D** | **R** | **K** | **E** | **Q** | **L** | **I** | **F** | **Y** | **W** | **M** | **C** | **H** |
| --- | --- | --- | --- | --- | --- | --- | --- | --- | --- | --- | --- | --- | --- | --- | --- | --- | --- | --- | --- |
| **A** | 4.5 | 5.3 | 5.2 | 5.4 | 4.9 | 4.7 | 4.7 | 4.9 | 5.0 | 5.1 | 4.9 | 4.7 | 4.9 | 5.1 | 4.6 | 4.3 | 4.9 | 4.9 | 4.7 |
| **V** |  | 5.6 | 5.5 | 5.5 | 5.2 | 5.5 | 5.5 | 5.6 | 5.7 | 5.6 | 5.5 | 5.4 | 5.5 | 5.6 | 5.5 | 5.4 | 5.4 | 5.3 | 5.2 |
| **P** |  |  | 5.6 | 5.7 | 5.0 | 5.3 | 5.3 | 5.8 | 5.7 | 5.5 | 5.6 | 5.3 | 5.3 | 5.4 | 5.5 | 5.1 | 5.3 | 5.2 | 5.3 |
| **T** |  |  |  | 5.4 | 5.2 | 5.4 | 5.3 | 5.7 | 5.7 | 5.6 | 5.5 | 5.4 | 5.5 | 5.6 | 5.3 | 5.5 | 5.4 | 5.3 | 5.4 |
| **S** |  |  |  |  | 5.4 | 5.3 | 5.2 | 5.2 | 5.2 | 5.1 | 5.2 | 5.3 | 5.5 | 5.3 | 5.6 | 5.4 | 5.4 | 5.4 | 5.3 |
| **N** |  |  |  |  |  | 5.5 | 5.6 | 5.7 | 5.6 | 5.4 | 5.5 | 5.5 | 5.7 | 5.6 | 5.8 | 5.9 | 5.8 | 5.3 | 5.6 |
| **D** |  |  |  |  |  |  | 5.5 | 5.6 | 5.5 | 5.6 | 5.6 | 5.6 | 5.7 | 5.7 | 6.1 | 5.8 | 5.6 | 5.4 | 5.7 |
| **R** |  |  |  |  |  |  |  | 6.3 | 5.8 | 5.8 | 5.8 | 5.7 | 5.6 | 5.9 | 5.9 | 5.6 | 5.5 | 5.2 | 5.6 |
| **K** |  |  |  |  |  |  |  |  | 5.7 | 5.6 | 5.8 | 5.5 | 5.5 | 5.7 | 5.6 | 5.6 | 5.6 | 5.4 | 5.6 |
| **E** |  |  |  |  |  |  |  |  |  | 5.7 | 5.7 | 5.5 | 5.6 | 5.8 | 5.8 | 5.7 | 5.7 | 5.1 | 5.5 |
| **Q** |  |  |  |  |  |  |  |  |  |  | 5.7 | 5.4 | 5.6 | 5.7 | 5.7 | 5.4 | 5.6 | 5.2 | 5.5 |
| **L** |  |  |  |  |  |  |  |  |  |  |  | 5.6 | 5.7 | 5.6 | 5.8 | 5.5 | 5.6 | 5.3 | 5.6 |
| **I** |  |  |  |  |  |  |  |  |  |  |  |  | 5.8 | 5.6 | 5.7 | 5.7 | 5.7 | 5.5 | 5.7 |
| **F** |  |  |  |  |  |  |  |  |  |  |  |  |  | 5.9 | 5.8 | 5.9 | 5.6 | 5.1 | 5.5 |
| **Y** |  |  |  |  |  |  |  |  |  |  |  |  |  |  | 6.1 | 6.0 | 5.7 | 5.6 | 6.2 |
| **W** |  |  |  |  |  |  |  |  |  |  |  |  |  |  |  | 6.2 | 5.6 | 5.1 | 6.0 |
| **M** |  |  |  |  |  |  |  |  |  |  |  |  |  |  |  |  | 5.5 | 5.5 | 5.7 |
| **C** |  |  |  |  |  |  |  |  |  |  |  |  |  |  |  |  |  | 5.4 | 5.2 |
| **H** |  |  |  |  |  |  |  |  |  |  |  |  |  |  |  |  |  |  | 5.5 |

**S3 Table. Inner well diameters.** Inner well diameters (Å) between side-chain centroids which are estimated from 711 PDBs with heavy atom distance cutoff 4.5Å. For outer well diameters, see the supplemental table 3 of the reference.[38]
