# Supplementary material for: Structural Conversion of Aβ17–42 Peptides from Disordered Oligomers to U-Shape Protofilaments via Multiple Kinetic Pathways
Source: PLoS Comput Biol. 2015 May 8;11(5):e1004258. doi: 10.1371/journal.pcbi.1004258 (PMC4425657; doi:10.1371/journal.pcbi.1004258)
Supplement: S4 Table — We multiply the original 19 parameters (Table III in reference [38]) by 1.3 to get the energy for the inner deep well and by 0.7 to get the energy for the outer shallow well. The salt-bridge between K and D is enhanced as -0.4×1.3 for the inner deep well and -0.4×0.7 for the outer shallow well. For detail classifications of parameters, please see reference [38]. (DOC) [file pcbi.1004258.s017.doc]

| **19 independent parameters for pairwise interactions in PRIME20** | | **Descriptions for Hydrophobic (HP) or Charge,  Size or Types of Amino Acids(A.A.) Hydrogen-Bond (HB) types** | **Pairwise interaction strength of** | **Pairwise interaction strength of** |
| --- | --- | --- | --- | --- |
| **Parameter names** | **Indices** |  | **inner deep well** | **outer shallow well** |
| NHCO | 1 | Backbone HB (ε_HB_) | -1.000 | -1.000 |
| LL,LM,MM | 2 | Strong HP, Small-Small A.A. | -0.200x1.3 | -0.200x0.7 |
| L{FYW},MF | 3 | Strong HP, Small-Large A.A. HB with S | -0.203x1.3 | -0.203x0.7 |
| M{YW} | 4 | Strong HP, Small-Large A.A. | -0.210x1.3 | -0.210x0.7 |
| F{FYW},WW | 5 | Strong HP, Large-Large A.A. | -0.205x1.3 | -0.205x0.7 |
| Y{Y,W} | 6 | Strong HP, Large-Large A.A. Side HB | -0.201x1.3 | -0.201x0.7 |
| AA | 7 | Weak HP | -0.084x1.3 | -0.084x0.7 |
| CC(Covalent Bond) | 8 | Breakable disulfide-bond | -0.585x1.3 | -0.585x0.7 |
| {ED}{ED} | 9 | Charged(--) | 0.253x1.3 | 0.253x0.7 |
| {E}{KR}, DR | 10 | Charged(-+) | -0.136x1.3 | -0.136x0.7 |
| {KR}{KR} | 11 | Charged(++) | 0.073x1.3 | 0.073x0.7 |
| A{LMFYW} | 12 | Weak HP, Ala- HP sidechains | -0.148x1.3 | -0.148x0.7 |
| C{LMFAC} | 13 | Cys-HP sidechains | -0.139x1.3 | -0.139x0.7 |
| C{YW} | 15 | Cys-HP sidechains, Side HB with S | -0.116x1.3 | -0.116x0.7 |
| {LMF}{EP},{YWC}P,{LF}S | 14 | HP sidechains – Small Polar(P) | 0.015x1.3 | 0.015x0.7 |
| {LF}{KQH},WK | 14 | HP sidechains – Large P | 0.015x1.3 | 0.015x0.7 |
| {MC}{KSQH},CE | 15 | Side HB with S | -0.116x1.3 | -0.116x0.7 |
| {YW}{ES},{ES}{SQH},KS | 16 | Side HB | -0.086x1.3 | -0.086x0.7 |
| {YW}{QH},YK | 16 | HP sidechains – Large P, Side HB | -0.086x1.3 | -0.086x0.7 |
| A{EKPSQH} | 17 | Ala - P | 0.074x1.3 | 0.074x0.7 |
| P{EKPSQH} | 17 | Pro - P | 0.074x1.3 | 0.074x0.7 |
| QQ,QH,HH | 18 | Large P – Large P, Side HB | -0.080x1.3 | -0.080x0.7 |
| Backbone-SideChain HB | 19 | Backbone-Side HB | 0.000 | 0.000 |

**S4 Table.** **19 independent energy parameters for double well potentials.** We multiply the original 19 parameters (Table III in reference [38]) by 1.3 to get the energy for the inner deep well and by 0.7 to get the energy for the outer shallow well. The salt-bridge between K and D is enhanced as -0.4×1.3 for the inner deep well and -0.4×0.7 for the outer shallow well. For detail classifications of parameters, please see reference [38].
